# CD98hc marks a precursor of human stem cell-derived alpha cells primed for functional maturation

**DOI:** 10.3389/fendo.2026.1923474

**Published:** 2026-09-28

**Authors:** Sean Lewis-Brinkman, Tiana Salomon, Kyle R. Knofczynski, Caden Duffy, Quinn P. Peterson

**Affiliations:** 1 Mayo Clinic Graduate School of Biomedical Sciences, Mayo Clinic, Rochester, MN, United States; 2 Department of Physiology and Biomedical Engineering, Mayo Clinic, Rochester, MN, United States

**Keywords:** enrichment, glucagon, human pluripotent stem cells, islet, maturation, stem cell-derived alpha cell (SC-α cells), type 1 diabetes

## Abstract

**Introduction:**

Pluripotent stem cell-derived alpha cells (SC-α cells) represent a promising component of next-generation cell therapies designed to more fully recapitulate human islet function; however, heterogeneity and the presence of undesired side populations limit their functional maturity and therapeutic potential. Here, we identify the cell surface protein CD98hc (SLC3A2), that when highly expressed in precursor cells, is associated with differentiation toward a more transcriptionally and functionally mature population of human SC-α cells.

**Methods:**

Directed differentiation was used to generate SC-α cells from pluripotent stem cells, which were assessed via single cell RNA sequencing, flow cytometry, and immunofluorescence imaging. Following magnetic activated cell sorting (MACS) of CD98hc enriched precursors, SC-α cell function was assessed using glucose stimulated glucagon secretion (GSGS).

**Results:**

We show that CD98hc is upregulated in SC-α cells expressing high levels of genes known to play important roles in α cell identity and function, and that CD98hc expression is elevated at the pre-α precursor stage relative to non–α cell populations. We demonstrate that magnetic enrichment of CD98hc⁺ pre-α cells derived from multiple pluripotent stem cell lines selectively increases the proportion of the highest CD98hc expressing glucagon-copositive precursors while depleting multiple undesired side populations, including somatostatin-positive and SOX9-positive cells. Although total α cell yield equalizes following complete differentiation, SC-α cells derived from high-CD98hc enriched precursors exhibit significantly enhanced glucose-responsive glucagon secretion compared to sham-sorted controls, indicating improved functional maturation of this precursor population.

**Discussion:**

Together, these findings establish that high expression levels of CD98hc marks a functionally superior α cell precursor, that enrichment at the precursor stage can bias differentiation toward higher functional α cell output, that stem cell-derived α cell populations harbor intrinsic functional heterogeneity, and that selective targeting of precursor populations represents a generalizable strategy to improve the quality and performance of stem cell-derived endocrine cell products.

## Introduction

Type I diabetes (T1D) is an autoimmune disease that results in the destruction of insulin-producing pancreatic β cells that affects approximately 8.7 million individuals worldwide, a number that is estimated to increase to 17.4 million by 2040 (1, 2). There is currently no cure for the disease, and individuals with T1D have a projected lifespan of approximately 12 years less than healthy counter parts (2, 3). Acutely, individuals with T1D are completely reliant upon exogenous insulin for survival (2). Chronically, higher blood glucose levels lead to an increased prevalence of micro- and macrovasculature damage, leading to complications such as cardiovascular disease, renal failure, neuropathy, and stroke (4).

Given the limitations of pharmacological management of T1D, the use of cell-based therapies that restore normal endocrine control of glucose homeostasis has been an area of focus for the past several decades. Transplantation of donor-pancreatic islets to T1D patients can result in durable insulin independence, with dramatic improvement in markers of glycemic control. Unfortunately, the quantity of available donor organs is insufficient to treat even the currently eligible T1D population, and the number of patients is expected to continue to expand (5, 6). Thus, there is an urgent need for more abundant sources of pancreatic endocrine cells for the treatment of diabetic patients.

Pluripotent stem cell-derived beta cells (SC-β cells) are a promising solution to the shortage of transplantable islets necessary to treat current and future individuals with T1D. Indeed, recent clinical studies have validated the ability of these cell products to produce clinical glycemic control (7). Nonetheless, significant interest remains in the development of improved SC-derived islet products. Multiple groups have developed differentiation protocols to generate SC-β cells that resemble primary human β cells in terms of morphology, gene expression, and function (8–10). Importantly, several studies have demonstrated the presence of subpopulations of SC-β cells with different functional properties (11–14). In an effort to generate more mature SC-β cells, methodologies have been developed to separate or enrich these functionally mature β cell populations (13, 15, 16).

Given the successes in developing protocols to generate and enrich SC-β cells, the focus has expanded towards generating more complex cell products that include both α cells and β cells to more closely recapitulate the complex interactions between endocrine cell types in the islet. α cells are known to play an important role in islet regulation. Several groups have reported that islet β cells exhibit increased function when interacting with other endocrine cell types, including α cells (17–19). To this end, we recently developed a protocol for generating glucagon-producing, pluripotent stem cell-derived alpha cells (SC-α cells) from a polyhormonal precursor stage (pre-α cells). These SC-α cells have been shown to protect against hypoglycemia *in vivo*, as well as to respond appropriately to a variety of secretory stimuli *in vitro* (20, 21). Such cells may be used to study α cells *in vitro* and ultimately may be incorporated along with SC-β cells into cell therapies that more closely resemble human islets.

Following up on our previous work with SC-α cells and the existence of heterogeneity in SC-β cells, we sought to identify populations of SC-α cells and α cell precursors that exhibit increased functional maturation analogous to what has been described in SC-β, and islet β cells (11, 22, 23). It stands to reason that downstream therapeutic populations of SC-β cells mixed with other cell types such as SC-α cells would benefit from being composed of the most functional populations of endocrine cells. Here we report the presence of a precursor population capable of producing higher functioning SC-α cells within our broader SC-α cell population that are marked by increased expression levels of the amino acid transport chaperone CD98hc. Following analysis of our single cell RNA sequencing dataset, the SC-α cluster can be divided into “mature” and “immature” signatures based on higher or lower than median expression levels of genes known to play an important role in α cell identity and function. Further analysis revealed a cell surface protein that was upregulated in the transcriptionally mature SC-α cell subpopulation and was effective at increasing the percentage of pre-α cells when utilized in a magnetically activated cell sorting protocol. Further differentiation of these enriched precursors then produced a population of SC-α cells with enhanced glucose responsiveness. This data suggests that selecting for a specific subpopulation of pre-alpha cells, even at the precursor stage, can have a functional impact on the final cell population. These results demonstrate that heterogenous stem cell-derived populations have a spectrum of functionality and support the development of a method to select for the most functional subpopulation using a cell-surface marker-based approach. This methodology may be applicable to other heterogenous stem cell-derived populations, and aid in producing more defined and functional cells for therapeutic purposes.

## Results

### Side populations are present in pre- and SC-α cell clusters

Toward the goal of identifying a functionally mature α cell population in our SC-α cell differentiation, we first sought to characterize the cell populations generated in our previously reported protocol (20). Immunofluorescent staining and flow cytometry analysis reveal the presence of both endocrine and non-endocrine cell populations (**Figures 1A, B**; **Supplementary Figure 1A**). Non-endocrine side populations were characterized at the pre-α and SC-α stages for delta-like cells (marked by somatostatin (SST)), proliferative cells (marked by Ki67), and immature pancreatic/ductal cells (marked by SRY-box transcription factor 9 (SOX9)). The latter two populations have the potential to be dangerous in a transplant setting, as they may lead to outgrowths from the engrafted cells. Our immunofluorescence analyses confirmed the presence of all three of these alternative cell types at both the pre-α and SC-α stages of the differentiation (**Figure 1B**). As previously reported (20), pre-α cells frequently co-express insulin (detected as C-peptide) (C-peptide^+^/Glucagon^+^), and resolve to monohormonal glucagon expression upon maturation into SC-α cells (C-peptide^-^/Glucagon^+^). Consistent with this report, following the presence of C-peptide^+^/Glucagon^+^ cells at the pre-α stage, there is a decrease of this cell type and an increase in C-peptide^-^/Glucagon^+^ cells at the SC-α cell stage. Together, these findings demonstrate that the SC-α differentiation protocol produces monohormonal, glucagon^+^ SC-α cells, as well as several undesired side populations.

**Figure 1:**
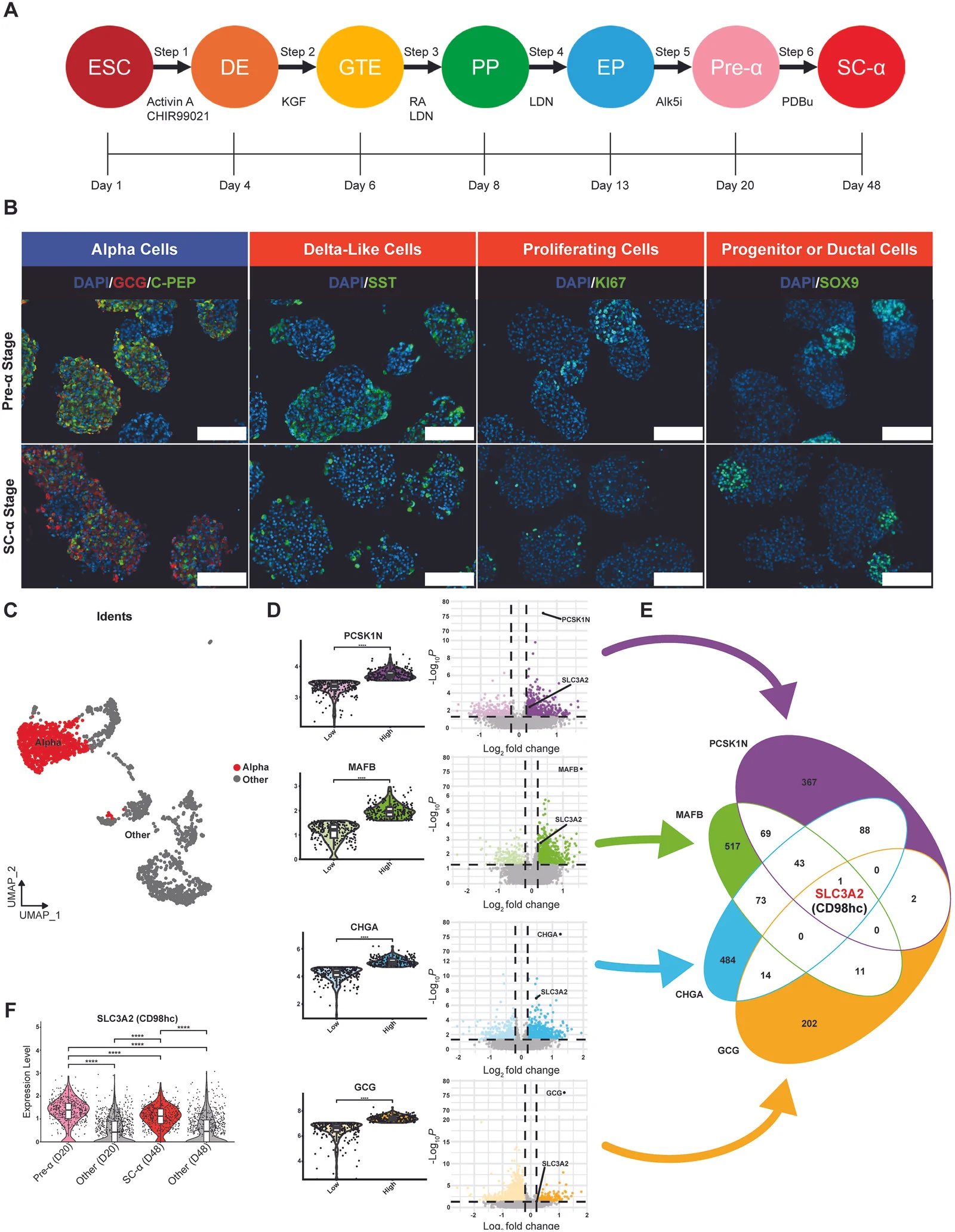
Identification of cell surface protein associated with-a cell maturity: **(A)**. Schematic timeline of the SC-α cell directed differentiation protocol. **(B)**. Immunofluorescent images of pre- α (top) and SC-α (bottom) cell clusters stained for the indicated markers of known side populations (scale bar=100μm). **(C)**. UMAP plot of single cell RNA sequencing data from pre- and SC-α cells. Alpha cells (α cells) are highlighted in red; all other cells regardless of identity are shown in grey and are collectively referred to as “other”. **(D)**. (Left) Violin plots showing “Alpha” cluster cells with higher and lower than median levels of expression of key α cell genes GCG, MAFB, CHGA, and PCSK1N (analyzed by Wilcox t-test, and Holm-Bonferroni multiple comparisons adjustment method, ****p<0.0001). (Right) Differential expression analyses performed on “high” expressors relative to “low” expressors of each of the four key α cell genes separately (color coded). Surface marker SLC3A2 appears on all volcano plots within log2fold change>0.2 and P-value<0.05. **(E)**. Venn diagram of differential expression analyses from panel D (right) displaying genes that are shared between different subgroups based on log2fold change>0.2 and P-value<0.05. Notably, SLC3A2 is the only gene shared by all four groups. **(F)**. Violin plot of transcript abundance of SLC3A2 (CD98hc) in pre-α (D20) stage cells, other cells at that stage, SC-α (D48) stage cells and other cells at that stage (analyzed by Wilcox t-test, and Holm-Bonferroni multiple comparisons adjustment method, ****p<0.0001).

Single-cell RNA sequencing of populations produced by our SC-α cell differentiation protocol (generated previously (20)) at the pre- and SC-α stages corroborate the identity of side populations at both of these stages of differentiation (**Figure 1C**; **Supplementary Figure 2A**). Approximately 20% of cells in this analysis are identified as α cells based on the expression of glucagon (GCG), transthyretin (TTR), Iroquois homeobox 2 gene (IRX2), and other α cell identity markers (**Supplementary Figures 2A, B**). The remaining cells (identified as “Other”, **Figure 1C**) include all side populations. The relative proportion of alpha cluster cells vs. other cell types is similar in the pre- and SC-α stages suggesting a maturation of the desired pre-α cell population rather than an expansion of the α cell population (**Supplementary Figure 2C**).

### The cell surface marker CD98hc marks a transcriptionally mature population of SC-α cells

Having demonstrated that both α cells and non-endocrine side populations were present at both the pre- and SC-α cell stages of the differentiation, we hypothesized that the α cells generated in our SC-α differentiation protocol were a heterogenous population with subpopulations of varying degrees of functional maturation. As such, we were motivated to find a surface protein that would enable identification and separation of the most functional α cell population from non-functional cells and side populations. Towards the goal of identifying the highest functioning SC-α cells within the larger SC-α cell population, we utilized the same single cell RNA sequencing data from above. Cells identified as alpha cells were first divided based on median expression levels of glucagon (GCG). Then differential expression analysis on the higher than median level GCG expressing alpha cluster subpopulation (229 cells) and the lower than median level GCG expressing alpha cluster subpopulation (230 cells) (**Figure 1D**) was performed. The process of comparing the differentially expressed genes of subpopulations of the same alpha cluster in our data set was repeated for other genes that are known to play an important role in α cell identity and function (GCG, MAFB, CHGA, PCSK1N) (**Figures 1C, D**, **Supplementary Figure 2D**). The differential expression analyses between higher and lower than median expression levels of the α cell genes generated four separate differentially expressed gene lists that represented genes associated with increased α cell identity (**Figure 1D** right panels). We next wanted to identify if there were any genes that were shared between the four subpopulations of upregulated genes. Overlapping genes between the four separate differentially expressed gene lists were filtered with gating parameters of log2 fold change>0.2 and P-value<0.05. Of the 1871 differentially expressed genes identified by this analysis, only one gene met these gating criteria and was shared in all four gene lists, the cell surface protein CD98hc (also known as SLC3A2, 4F2h or 4F2hc) (**Figure 1E**). CD98hc is a heterodimeric protein which enables amino acid transport via association with a variety of light chains (24). We initially hypothesized that increased expression of this gene describes a more transcriptionally mature subpopulation of SC-α cells and would be a suitable candidate for enrichment of functionally mature SC-α cells. The selection of CD98hc was supported by the observation that levels of CD98hc expression were elevated in pre-α cells relative to the other cells in the pre-α stage data. This suggests increased levels of CD98hc expression could be used to enrich for either SC-α or pre-α cell precursors (**Figure 1F**).

To determine when during the directed differentiation protocol our protein of interest CD98hc began to be expressed and to gauge its co-expression at the protein level with glucagon-expressing pre- and SC-α cells, immunofluorescence staining and flow cytometry were performed at multiple stages throughout the SC-α cell directed differentiation protocol. Surprisingly, CD98hc is expressed throughout the entirety of the directed differentiation protocol (**Figure 2A**). At the early stages of the differentiation protocol, nearly all cells express CD98hc. However, the expression levels of CD98hc notably increase in the glucagon co-expressing cell population to yield distinct “low-CD98hc” and “high-CD98hc” subpopulations during the pre- and SC-α cell stages (**Figures 2B, C**, **Supplementary Figure 3**). We also observed that greater than 70% of cells during both the pre- and SC-α stages expressed CD98hc on the cell surface, as assessed via live cell staining for CD98hc (**Supplementary Figures 4A, B**). At the same time, CD98hc expressing cells that do not co-express glucagon (CD98hc^+^/Glucagon^-^) decrease in abundance over the course of the differentiation, while CD98hc expressing cells that co-express glucagon (CD98hc^+^/Glucagon^+^) increase (**Figure 2D**). Together these findings demonstrate that CD98hc colocalizes with the glucagon positive cell population on the protein level, is expressed on the cell surface, and is expressed at higher levels in the glucagon co-positive subpopulation, increasing in population percentage over the course of the differentiation.

**Figure 2:**
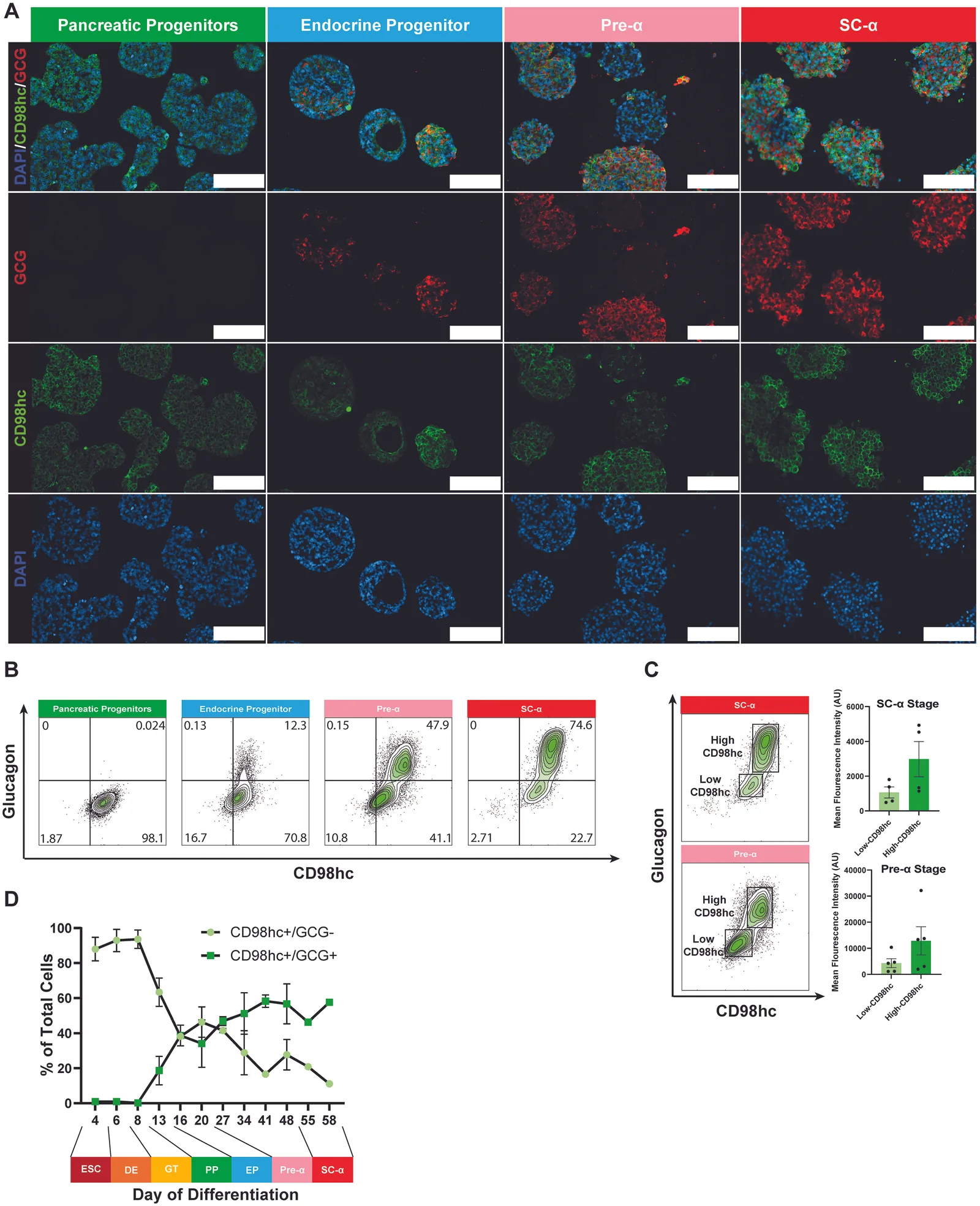
Cell surface marker CD98hc is upregulated on pre- and SC-α cells: **(A)**. Immunofluorescent images of cell clusters stained for glucagon (red), CD98hc (green), and nuclei (blue) at the indicated stages of the SC-α cell differentiation protocol (scale bar=100µm). **(B)**. Flow cytometry analysis of cells collected at the indicated stages of the SC-α cell differentiation protocol stained for CD98hc and glucagon. **(C)**. (Left) Representative flow cytometry of pre- and SC-α stage cells stained for CD98hc and glucagon demonstrating the presence of “high-CD98hc” and “low-CD98hc” subpopulations (n=7 independent experiments). (Right) mean fluorescence intensity (MFI) values of the low- and high-CD98hc populations at their respective stages, further demonstrating that there is a difference in CD98hc expression level between the two subpopulations (n=4 and n=5 independent experiments). **(D)**. Summary plot of multiple flow cytometry experiments (n=7 independent experiments) showing population percentages of CD98hc^+^/Glucagon^-^ and CD98hc^+^/Glucagon^+^ cells throughout the SC-α cell differentiation. Data are represented as mean ± standard error of the mean.

### Enrichment is effective for pre-α cells and depletes cells lacking glucagon expression

Having identified high levels of CD98hc expression as a putative marker of transcriptional maturation in SC-α cells, we designed a magnetically activated cell sorting (MACS) protocol to preferentially select for cells with highest levels of CD98hc expression (25) (**Figure 3A**). Briefly, clusters of cells from the pre-α or SC-α cell stages (day 20–34 or >47 respectively) of the SC-α directed differentiation protocol were dissociated, incubated with primary anti-CD98hc antibody, followed by a magnetically conjugated secondary antibody, and then passed over a MACS columns. The flow-through fraction was collected first along with rinses from the column, and then the MACS columns were removed from the magnetic field to elute the CD98hc-sorted or sham-sorted fraction (depending on the presence or absence of anti-CD98hc and magnetically conjugated antibodies respectively). The collected fractions of single cell solutions were then reaggregated over the course of 4 days using 384 well spheroid plates, and the resultant clusters were transferred to 6 well plates for continued culture until terminally differentiated (Day 47 and onwards) (**Figure 3A**).

**Figure 3:**
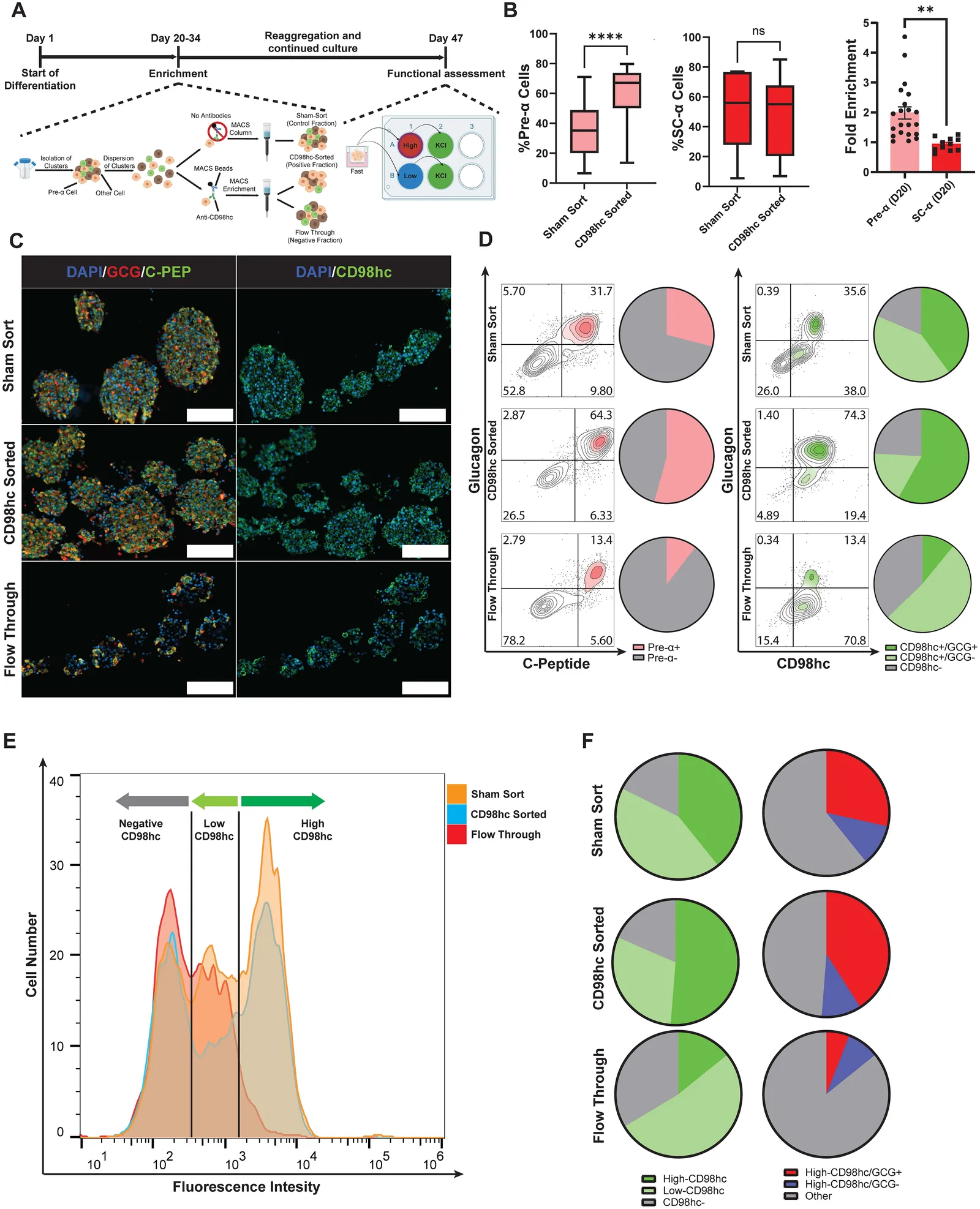
MACS for CD98 at the pre-α cell stage depletes cells lacking glucagon expression: **(A)**. Timeline of enrichment procedures and functional assessment of SC-α cells. **(B)**. Percentage of pre- or SC-α cells immediately following enrichment, as identified by flow cytometric staining for pre-α cells (C-peptide^+^/Glucagon^+^) during the pre-α stage, and SC-α cells (C-peptide^-^/Glucagon^+^) during the SC-α stage, compared to sham-sorted controls (n=21 independent experiments at pre-α stage, and n=11 independent experiments at SC-α stage) first two panels analyzed by paired t-test ****p<0.0001 and third panel analyzed by unpaired t-test **p<0.001. **(C)**. Immunofluorescent images of reaggregated cell clusters derived from CD98hc-based enrichment stained for pre- or SC-α cell identity (Glucagon^+^/C-peptide^+^ or Glucagon^+^/C-peptide^-^ respectively) and cell surface marker CD98hc (enriched on D20 and stained following reaggregation on D26) (scale bar=100µm). **(D)**. Representative flow cytometry of fractions derived from sham-sorted (top), CD98hc-sorted (middle) or flow-through (bottom) cell populations during step 6 of the differentiation protocol, stained for C-peptide and glucagon (left), or CD98hc and glucagon (right). Pie charts summarizing multiple independent differentiations, enrichments and flow cytometry experiments on the right (n=7 independent experiments). **(E)**. Representative gating strategy to quantify proportions of cells with high, low, and negative CD98hc expression. **(F)**. Pie charts summarizing the proportion of cells with high, low, and negative CD98hc expression (left), and the proportion of cells with high CD98hc and glucagon co-expression (right) in enrichments conducted during step 6 of the differentiation protocol (n=4 independent experiments).

Given the observation that CD98hc expression was increased in glucagon expressing cells at both the pre- (Day 20-34) and SC-α (Day 47+) cell stages, enrichment was performed at both of these stages (**Figure 2C**). Importantly, we did not observe enrichment of fully differentiated SC-α cells (C-peptide^-^/Glucagon^+^) at the SC-α stage (Day 48 and onwards) (average of 49.1 ± 7.7% SC-α cells in sham sort and 46.6 ± 8.5% SC-α cells in CD98hc-sorted), but surprisingly observed an approximate two-fold enrichment of pre-α cells (C-peptide^+^/Glucagon^+^) relative to the sham-sort group at the pre-α stage (day 20-34) (average of 36.7 ± 4.1% pre-α cells in sham sort and 60.1 ± 3.9% pre-α cells in CD98hc-sorted), as determined by flow cytometry analysis (**Figure 3B**). This enrichment at the pre-α cell stage was verified in multiple differentiations from an alternative iPS cell line (**Supplementary Figure 5**). This enrichment result at the pre-α cell stage is consistent with the single cell RNA sequencing data, where SC-α cells had significantly lower levels (adjusted p-value= 1.8e^-12^) of CD98hc expression than pre-α cells and thus the differential expression of CD98hc in SC-α cells vs. other cells during the SC-α cell stage of the differentiation was not as pronounced (**Figure 1F**). Further corroborating this, the mean fluorescence intensity values of the high-CD98hc subpopulation at the pre-α stage (average of 12,859 ± 5,418) are four-fold higher than those at the SC-α stage (average of 2,983 ± 1,012) (**Figure 2C**). This results indicate that CD98hc is not marking a transcriptionally mature SC-α cell state, but may instead be marking a unique precursor population of pre-α cells.

To further characterize enrichment targeting CD98hc expression levels at the pre-α cell stage, immunofluorescence (**Figure 3C**) and flow cytometry (**Figure 3D**; **Supplementary Figure 6**) were performed using stains for C-peptide, glucagon, and CD98hc on reaggregated fractions obtained following CD98hc-based enrichment. These analyses verified the ~two-fold enrichment of pre-α cells observed previously (average of 29.1 ± 3.8% pre-α cells in sham sort fraction, 54.3 ± 7.8% pre-α cells in the CD98hc sorted fraction). Pre-α cells were conversely depleted in the flow-through fraction (average of 10.4 ± 3.3% pre-α cells in the flow through fraction) (**Figure 3D**). Interestingly, the proportion of CD98hc expressing cells was comparable in the sham sort fraction vs the CD98hc sorted fraction, although it was reduced in the flow through fraction (82.3 ± 6.3% in sham sort fraction, 81.5 ± 8.2% in CD98hc sorted fraction, and 66.4 ± 13.7% in flow through fraction) (**Figure 3D**). However, the proportion of glucagon-positive cells within the total CD98hc positive population was markedly higher in the CD98hc-sorted fraction vs the sham-sorted fraction (average of 54.1 ± 6.9% vs 38.9 ± 3.3% respectively) (**Figure 3D**). This data indicates that enrichment targeting CD98hc favors the glucagon-expressing precursors within the total CD98hc expressing population of cells. In addition to the observed enrichment of glucagon expressing cells, a decrease in the side populations previously described in **Figure 1B** was observed suggesting that sorting also eliminates unwanted cell types. Further investigation of the fractions obtained through enrichment via immunofluorescence and flow cytometry revealed a decrease in somatostatin+/glucagon- cells, and SOX9+ cells, however, there was no observable depletion of Ki67+ cells (**Supplementary Figures 7A, B**). This importantly demonstrated that enrichment, in addition to increasing pre-α cells, also has the ability deplete some of the previously observed side populations.

Similar to the previous observation that there are distinct low-CD98hc and high-CD98hc expressing subpopulations in the SC-α differentiation (**Figure 2C**), a more detailed analysis of the CD98hc flow cytometry data following enrichment revealed a similar pattern of CD98hc expression (**Figure 3E**). Further analyzing the cell fractions produced by our MACS protocol demonstrated that the protocol specifically enriches for the CD98hc-high population (**Figure 3F**, left panels). Additionally, there is a large portion of the CD98hc-high cells that co-express glucagon (**Figure 3F**, right panels). High-CD98hc/glucagon^+^ cells comprise 40.9 ± 3.9% of the CD98hc-sorted cell population, compared to only 28.5 ± 2.5% of the sham sort population. Low-CD98hc cells end up largely in the flow through population 52.2 ± 7.1%. This demonstrated that our enrichment protocol increases the population percentage of cells that have higher levels of CD98hc expression, and that cells with high CD98hc expression also tend to co-express glucagon, thus enabling an enrichment of glucagon producing precursor cells at the pre-α cell stage (**Figure 3F**, right panel). Further corroborating this, we found that following enrichment via CD98hc, the ratio of glucagon producing precursor cells with the highest expression levels of CD98hc relative to cells with moderate expression levels of CD98hc increased in the CD98hc-sorted fraction (**Supplementary Figures 8A–C**). This demonstrates that the CD98hc-sorted group shifted the total CD98hc^+^/Glucagon^+^ subpopulation to favor highest-CD98hc expressing precursors compared to the sham sort controls.

### SC-α cells derived from CD98hc enriched pre-α cells exhibit improved glucose responsivity

Increased expression levels of CD98hc are transcriptionally associated with more transcriptionally mature SC-α cells in our single cell RNA sequencing dataset, and we were able to demonstrate that we can select for highest-CD98hc expressing precursor cells via our enrichment protocol (**Supplementary Figures 8A–C**). As such, this highest-CD98hc enriched pre-α cell precursor population may be competent to produce a more transcriptionally mature or a more glucose responsive SC-α cell population. To test this, the CD98hc-sort derived pre-α cell fraction was cultured for the remainder of the SC-α cell differentiation protocol until terminally differentiated, characterized and functionally assessed (**Figure 3A**). Surprisingly, flow cytometry analysis at the SC-α stage (Day 47 and onwards) of the fractions following enrichment during the pre-α stage (day 20-34) demonstrated that the total population percentage of SC-α cells (C-peptide^-^/Glucagon^+^) was comparable between the two populations, at an average of ~45%. The proportion of total glucagon expressing cells (both C-peptide^-^/Glucagon^+^ and C-peptide^+^/Glucagon^+^) was also comparable (**Figure 4A**; **Supplementary Figures 9**, **10A**). Thus, despite the enrichment of glucagon^+^ cells by CD98hc sorting at the pre-α precursor stage, the proportion of glucagon expressing cells becomes more similar across cell populations during subsequent culture and differentiation. Importantly, we observe that CD98hc enrichment derived SC-α cells maintained significantly higher population percentages of high-CD98hc cells than their sham-sorted counterparts (p<0.05) (**Supplementary Figure 10B**).

**Figure 4:**
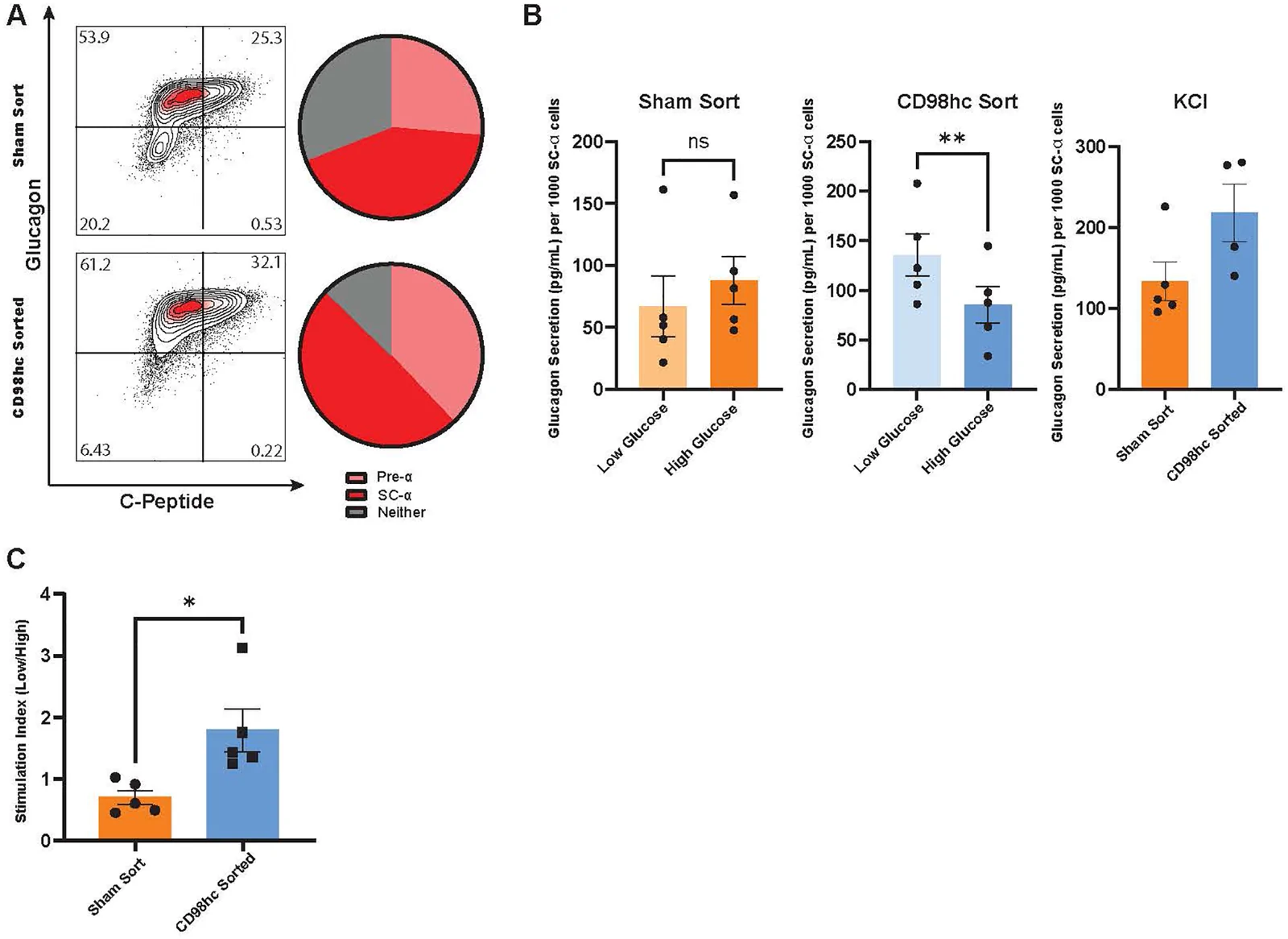
SC-α cells derived from CD98hc-sorted pre-α cells exhibit improved glucose responsivity. **(A)**. Flow cytometry of fractions derived from CD98hc-sorted pre-α cells that were further differentiated to the SC-α cell stage (day 48), showing population percentages of C-peptide^+^/Glucagon^+^ (pre-α cells), C-peptide^-^/Glucagon^+^ (SC-α cells), and C-peptide^-^/Glucagon^-^ (neither). Representative flow plots on the left and pie charts representing multiple flow cytometry experiments (n=4 independent experiments) on the right. **(B)**. Quantification of glucose stimulated glucagon secretion (GSGS) from sham- vs CD98hc-sorted pre-α cells that were further differentiated to the SC-α cell stage. Data were normalized to the number of SC-α cells (C-peptide^-^/glucagon^+^) using total cell count following the secretion assay, and flow cytometry of populations assessed (n=5 independent experiments) analyzed by paired t-test *p<0.05, **p<0.01. **(C)**. Stimulation index (glucagon secretion in low glucose divided by glucagon secretion in high glucose) as a measure of SC-α cell function for the cells analyzed in B (n=5 independent experiments) analyzed by paired t-test* p<0.05.

To evaluate the transcriptional maturity of the CD98hc enrichment derived SC-α cells, the expression levels of key genes known to play important roles in human islet α cell identity and function and that are known to change expression with transcriptional maturation (MAFB, ARX, IRX1) (26) were evaluated by qPCR (**Supplementary Figure 10C**). An increase in the global expression level of these maturation markers was not observed in this heterogenous population suggesting that transcriptional maturation may not be significantly improved in the CD98hc enriched precursor population although the insensitivity of this population level analysis does not preclude subtle transcriptional and pathway level changes that may impact the functional maturity of these cells.

To this end, the physiological implications of sorting for the CD98hc high expressing precursor cells was evaluated. *In vivo*, α cells secrete glucagon in response to hypoglycemia but suppress glucagon secretion under hyperglycemic conditions. To assess the physiological function of SC-α cells derived from CD98hc enriched precursors, SC-α cells were evaluated in a glucose stimulated glucagon secretion (GSGS) assay and compared to SC-α cells derived from sham cells. This assay subjects enrichment-derived SC-α cell fractions to both high (16.3 mM) and low (3.3 mM) glucose conditions, and glucagon secretion by the SC-α cells in response to these stimuli is measured by ELISA. Interestingly, CD98hc-sorted derived SC-α cells displayed a significant increase in glucagon secreted under low glucose than that under high glucose (p<0.01). In contrast, sham-sorted controls secreted a similar amount of glucagon in response to both low and high glucose, suggesting a greater sensitivity to low glucose conditions in CD98hc-sorted derived SC-α cells (**Figure 4B**). Additionally, enrichment-derived SC-α cells may have an increased secretory capacity for glucagon as demonstrated by a trending towards higher KCl response (**Figure 4B**). A stimulation index was calculated by dividing the glucagon secreted under low glucose (stimulatory condition) by the amount of glucagon secreted under high glucose (inhibitory condition). CD98hc-sorted derived SC-α cells demonstrated a statistically significant increase in stimulation index compared to sham sort derived SC-α cells (p < 0.05). Sham sort SC-α cells were dysfunctional (SI<1), while CD98hc enrichment generated functional SC-α cells (SI>1), and increased the SI by 2.5-fold (**Figure 4C**). Importantly, this data demonstrated that the SC-α cells derived from CD98hc-enriched pre-α cell precursors displayed a significant improvement in glucose responsivity compared to their sham-sorted counterparts, despite having similar proportions of SC-α cells, lending support to the claim that CD98hc enriched pre-α cells represent a precursor population competent to generate a more functional population of SC-α cells. Further, all secretion assays were normalized to the number of SC-α cells (C-peptide^-^/Glucagon^+^) present in the assay so that only the relationship between glucagon secreted under low and high glucose conditions was being evaluated (**Figures 4B, C**).

To further evaluate the transcriptional basis for the improved functional maturity of these SC-α cells derived from CD98hc-enriched pre-α cell precursors we performed a more sensitive transcriptional analysis was performed using single cell level and pathway level analysis. Analysis of our single cell transcriptional dataset (from **Figure 1C**), demonstrated an increase in these key α cell related pathways cells with higher level of CD98hc. Cells clustering with α cells transcriptional similarity were separated into high-CD98hc and low-CD98hc expressing populations and were then compared via pathway analysis (**Supplementary Figures 11A–C**). Several of the enriched pathways in this CD98hc high population are important in α cell maturity and function including amino acid metabolism and transport, circadian signaling and zinc transport. While bulk qPCR analysis of the heterogenous population (~45% SC-α cells) was insufficient to observe increased transcriptional maturation, the single cell transcriptional results suggest that the subpopulation of pre-α cell precursors selected via sorting on CD98hc give rise to SC-α cells with enhanced signaling pathways associated with α cell function. Taken together, these results suggest that enriching for the highest-CD98hc pre-α cell precursors results in a population of SC-α cells with improved functional maturity.

## Discussion

Here we have identified a cell-surface protein, CD98hc, that when highly expressed is effective for enriching a population of precursor (pre-α cells) that differentiate into functionally mature SC-α cells from a mixed cell population. CD98hc is the sole significant overlapping gene between high expressors of various genes important in α cell identity and function and is elevated in α cells more robustly expressing this α cell transcriptional signature. Further analysis using single-cell RNA sequencing data revealed that that CD98hc (SLC3A2) is expressed at a higher level in pre- and SC-α cells relative to the other cells present during those stages in the differentiation. We show that high expression levels of CD98hc can be utilized in conjunction with magnetically activated cell sorting (MACS) to enrich pre-α cells by approximately two-fold at the pre-α cell stage (27). Enrichment was not observed when sorting was performed at the SC-α cell stage, likely due to lower levels of differential CD98hc expression between SC-α and other cells at the SC-α stage than pre-α cells and the other cells at the pre-α stage. Further differentiation of CD98hc-enriched pre-α cells into SC-α cells resulted in a population with 2.5-fold increased glucose responsive glucagon secretory function compared to sham-sorted control cells. This indicates that pre-α cells with higher levels of CD98hc expression relative to other cells at the pre-α stage give rise to a more functional subpopulation competent to differentiate into a more functional SC-α cell population that more strongly express key signaling pathways exhibited in primary human α cells and thus may be more amenable to development as part of a cell-based therapy for diabetes.

CD98hc is known to be expressed in human islets, specifically α and β cells (28), confirming that our observation of its presence in SC-α cells is consistent with primary human tissue. CD98hc is a heavy-chain glycoprotein (4F2hc encoded by SLC3A2) with a molecular weight of 80-85kDa and associates with a variety of light chains (29–31), forming complexes that act as amino acid transporters (24). Our single cell RNA sequencing dataset shows that both CD98hc and LAT2 (SLC7A8) are upregulated in SC-α cells in comparison to all other cell types during the SC-α stage (log2 fold change values of 0.96 and 2.05 respectively). LAT2 when bound to CD98hc facilitates the transport of multiple amino acids, including alanine, serine, and threonine (31).

Recently, α-cells have been noted for their role in amino acid sensing and metabolism (32–35), suggesting that the expression of CD98hc may be of functional importance. Several amino acids have been shown to stimulate glucagon secretion, suggesting that coupling glucagon secretion to ambient amino acid levels is of functional importance. (36–38) For example, it has been suggested that an important physiological role of glucagon may be to stimulate amino acid uptake and processing by the liver in conditions of amino acid excess (39, 40). It may be that higher expression of CD98hc and LAT2, as observed in SC-α cells, enables improved amino acid sensing by these cells, reflecting a more mature α-cell like phenotype.

Additionally, it has been shown that following exposure to diabetogenic stressors in culture, SC-α cells have downregulated expression of amino acid transport proteins (Shrestha et al.). This further suggests that amino acid transport is an important dimension of α cell functional maturity. By selecting pre-α cells that have increased CD98hc expression in our protocol, the selection of a more receptive/competent precursor population that gives rise to more functional SC-α cells may be due in part to the role of amino acid transporters in alpha cell function. This possibility deserves further investigation and will be the subject of future studies.

Methods to purify specific pancreatic cell types from either primary islets or stem cell-derived populations have been the subject of extensive prior research. A wide variety of methods have been tested, including gradient-based separation (41, 42), cell surface markers (42, 43), fluorescent qualities (44, 45), adenovirus transfection (46), or zinc content (47). There are even methods that utilized metabolic state of islet cells (48) and proliferative status of cells to separate populations (49). Previous efforts have demonstrated that sorting SC-β cells increases their *in vitro* and *in vivo* functionality (15, 16, 25). More recent efforts demonstrate methods to yield SC-β cells with high purity of >60-80% using dual marker approaches (25, 50). These studies demonstrated a 2.5- and 3-fold enrichment of SC-β cells using MACS and FACS respectively. While not the focus of these studies, the approaches reported may conversely be used to increase the purity of alpha cells. While our methodology resulted in a 1.5- to 2-fold increase in glucagon positive cells at the time of sorting (pre-α cells), this enrichment for glucagon positive cells was not durable to later stages of differentiation. In contrast, our methodology is aimed at changing the functional state of the cell population, rather than simply increasing the purity of the cells generated. Notably, purity alone is not likely sufficient to improve the performance of cell products. For example, Malakandov et al. reported that enrichment of SC-β cells using CD49a (enriched to ~44% SC-β cells) did not provide therapeutic benefit over an unsorted populations of SC-β cells (~22.6% SC-β cells) (25). In another study, CD49a+/CD133+ sorted cells (~75% SC-β cells) was functionally indistinguishable from not only CD49a+ (~40% SC-β cells), but also unsorted SC-islets (~25% SC-β cells). These results highlight the limitation of sorting strategies that are focused on cell purity—these approaches are unable to distinguish between functionally mature and immature cells (25, 50). In contrast, our study identified a method to enrich for a competent pre-α cell precursor population produces functionally improved SC- α cell populations despite similar purity to the unsorted SC-α cells—notably, this enrichment approach does not fully purify pre-α cells, leaving approximately 60-70% non-pre-α cells. The approach described here has significant potential to be applied to other cell types and improve the production of functionally mature cell products. It is worth noting that the maturation competent precursor strategy used here is complementary to and may be employed in combination with the purity based enrichment approaches put forth by others. These combination studies will be reported in due course.

Here we have reported a methodology to preferentially enrich the functionally mature SC-α cell population by targeting the intermediate precursors, pre-α cells, in our directed differentiation protocol. Additionally, we have identified a novel association between more mature and functional SC-α cells to increased expression levels of the cell surface protein CD98hc. Our findings underscore the important concept of functional heterogeneity in stem cell-derived populations and demonstrate a methodology to isolate the most functional members of that population. Modulation of heterogeneous cell populations may be a critical aspect of making a maximally effective cell therapy.

## Methods

### Embryonic stem cell culture

The embryonic stem cell line HUES8 (NIHhESC-09-0021) and the iPS cell line 1016 were obtained from the HSCI iPS Core Facility (Harvard University). ES and iPSC cells were cultured and expanded in suspension culture as described previously (8). Spinner flasks (500mL volume, Corning, VWR, CLS3578) were seeded with 0.5 million cells/mL in 300 mL of mTeSR™ media (STEMCELL Technologies) supplemented with 10 µM Y27632 (Tocris) and maintained at 70 rpm in a humidified incubator at 37 °C and 5% CO_2_. After 48 hours, clusters were allowed to settle, spent media was removed by aspiration, and replaced with fresh mTeSR media without Y27632. Cells were passaged every 72 hours by using Accutase to disperse clusters into single cells and seeding them into fresh mTeSR media supplemented with 10 µM Y27632. Cell identity, sterility and mycoplasma status were evaluated every 10 passages by DNA fingerprinting, antibiotic free culture and evaluation for growth of bacteria and mycoplasma.

### Directed differentiation

Prior to differentiation, HUES8 cells were seeded into mTeSR at a density of 0.5 million cells/mL, and clusters were allowed to form for 72 hours. Clusters were then allowed to settle at the bottom of the spinner flask, and spent media was removed by aspiration allowing for a complete media change. Differentiation was conducted in the same spinner flask, maintained at 70 rpm in a humidified incubator at 37 °C and 5% CO_2_. Media and growth factors used were as described previously (8, 20) and summarized as follows: Day 1: S1 + 100 ng/mL Activin A (R&D Systems) + 3µM CHIR99021 (Stem Cell Technologies). Day 2: S1 + 100 ng/mL Activin A. Day 4: S2 + 50 ng/mL KGF (Reprotech). Day 6: S3 + 2µM RA (Tocris). Day 7: S3: + 2µM RA + 200nM LDN193189 (Tocris). Day 8: S3 + 200nM LDN193189. Days 9, 11: S3 (no additional factors). Days 13, 15, 17, 19: S3 + 10µM Alk5i (Tocris). Days 20-48 (fed on even numbered days only): S3 + 500µM PDBu (Tocris).

### MACS enrichment

Pre-α cell clusters (taken at day 20–34 or before) and SC-α cell clusters (taken at day 48 and onward) were dispersed into single cells using TrypLE at 37 °C, quenched with equal volume BSA-containing media, resuspended in sorting buffer (PBS + 2 mM EDTA + 1% fatty acid-free BSA) and passed through a 40µm filter (Pluriselect). The resulting single cell suspension (10 million cells per 300µL of sorting buffer) was then co-incubated with an anti-CD98 antibody (Invitrogen, MA1-19195) at a dilution of 1:1000 for 20 min on ice. The cells were then washed twice with 5 mL of sorting buffer, spun down at 200 x g for 5 min, and resuspended at the original density of 10 million cells per 300µL in sorting buffer. Anti-mouse IgG MACS microbeads (Miltenyibiotec, 130-048-401) were added at a concentration of 40µL MACS beads per 10 million cells for 15 min at 4 °C. The cell/microbead complexes were then washed twice as described above before being added to MACS LS columns (Miltenyibiotec 130-042-401) attached to a Quadro MACS separator (Miltenyibiotec, 130-042-303) at a density of no more than 30 million cells per 500µL, ensuring that each column received no more than 30 million cells. The cell solution was then washed through column with 3 mL of sorting buffer three times, and the flow through fraction was collected. MACS columns were then removed from the Quadro MACS separator, and 5 mL of sorting buffer was plunged through the LS columns and collected as the CD98-sort fraction. Sham-sorted control cells were handled identically, without addition of the anti-CD98 antibody. Collected fractions containing dispersed single cells were then reaggregated via plating in ultra-low attachment 384-well spheroid plates (Corning, 3830) at 20,000 cells per 50µL and incubated for 4 days. Once reaggregated, cells were plated in ultra-low attachment 6-well plates (Corning, 3471) and set on a rocker at 25 rpm in a humidified 37 °C and 5% CO_2_ incubator.

### Flow cytometry

Clusters of cells were collected at the indicated phases of the differentiation protocol and enrichment process. Clusters of cells were washed with PBS and then dispersed with TrypLE Express at 37 °C for 10 min after which the TrypLE was quenched with S3 (BSA-containing) media and mixed thoroughly with a p1000 pipette to mechanically disperse the remaining clusters. Cells were fixed in 4% paraformaldehyde (PFA) for at least 1 hour at room temperature (RT) or overnight at 4 °C. Fixed samples were stored in PBS at 4 °C until staining. Staining was performed by running fixed cells through at 40µm filter, washing once with PBS, washing once with PBST, and permeabilizing the cells in block solution (PBS + 0.1% Triton X-100 + 5% donkey serum) for 1 hour at RT. Cells were stained with primary antibodies in block solution for 1 hour at RT, washed twice with PBST and then stained with secondary antibodies in block solution for 1 hour at RT. Cells were then washed three times and resuspended in PBST before being analyzed using an Attune NXT flow cytometer. Exported FCS files were analyzed using FlowJo version 10.10.1.

### SC-RNA sequencing

Single-cell RNA sequencing data was generated previously and available in the Gene Expression Omnibus under the accession number GSE138857 (20). Cells with less than 500 detected genes, more than 25% mitochondrial content, or less than 50,000 RNA transcripts were removed from the data before normalization and integration. Data was analyzed using the Seurat (v.5.1.0) (51). UMAP was used for dimensionality reduction based on the first 30 dimensions. Individual clusters were manually identified based on the most differentially expressed and upregulated genes present in the cluster. Venn diagram was generated using ggVennDiagram (52).

### Immunofluorescence

Clusters were fixed in 4% PFA for 1 hour, washed with PBS, and embedded in Histogel before being subsequently embedded in paraffin and cut into 4µm sections. For immunostaining, the sectioned samples were deparaffinized by serial incubation (Toulene-10 min; Toulene-10 min; 100% Ethanol-10 min; 95% Ethanol-5 min; 70% Ethanol-5 min; Distilled Water-3 min). Antigen retrieval was performed by boiling slides in sodium citrate buffer at pH 6.5 for 1 hour, followed by cooling to room temperature for at least 45 minutes. Samples were then incubated with blocking solution in a humidified chamber for 1 hour at room temperature, followed by incubation with primary antibodies for 1 hour at room temperature. After five washes with PBST, slides were incubated with secondary antibodies in blocking solution with DAPI (4′,6-diamidino-2-phenlindole) for 1 hour at RT and then rewashed with PBST (**Supplementary Table 2**). Slides were mounted using Fluoromount-G and imaged and analyzed with Zeiss Widefield Microscopy and Zen2 software. The area of each section and cell counts were quantified using Zen software. Cell counts were normalized to the total area of the stained section.

### Reverse transcriptase quantitative polymerase chain reaction

Frozen cell clusters were thawed on ice, and RNA was extracted using Qiagen RNeasy^®^ Mini Kit per the manufacturer’s instructions. RNA quantification was performed using NanoDrop™ One spectrophotometer (Thermo Scientific). RNA with 250/280 ratio of 1.8-2.2 were used for experiments. cDNA was generated from 400ng of RNA using a high-capacity reverse transcriptase kit (iScript cDNA synthesis kit, Bio-Rad). Quantitative PCR (qPCR) reactions were performed in a Roche Lightcycler^®^96 and utilized SYBR green PCR master mix (Applied Biosystems) and primers obtained through Integrated DNA Technologies with sequences listed in **Supplementary Table 1**. Gene expression was analyzed using the 2^-ΔΔC_t_ method.

### Glucose stimulated glucagon secretion assay

Cell clusters were washed twice in high glucose (16.3 mM) Krebs-Ringer buffer (KRB; 128 mM NaCl, 5 mM KCl, 2.7 mM CaCl_2_, 1.2 mM MgSO_4_, 1 mM Na_2_HPO_4_, 1.2 mM KH_2_PO_4_, 5 mM NaHCO_3_, 10 mM HEPES (4-(2-hydro-xyethyl)-1-piperazineethanesufonic acid), 0.1% BSA in MilliQ H_2_O). Cell clusters were loaded into 24-well ultra-low attachment plates (Corning, cat no. 3473) with transwell inserts (Millicell, cat no. PIXP01250) and pre-incubated in high-glucose KRB (16.3 mM) for 1 hour at 37°C on a plate rocker. Cell clusters were then transferred to either high-glucose KRB or low-glucose KRB (3.3 mM) in a parallel secretion assay for 1 hour at 37 °C on a plate rocker. Cell clusters were then incubated in low-glucose KRB (3.3 mM) with 30 mM KCl for 1 hour at 37 °C on a plate rocker. Supernatants from all conditions were collected after incubation step and stored at -20 °C until analysis. Cell clusters were then dispersed by incubating in TrypLE Express for 20 min at 37 °C on a plate rocker, followed by thorough mixing with a p1000. Dispersed cells were then counted using Trypan Blue and a Cell Countess II FL (Invitrogen: 1.0.24). Collected supernatants were analyzed by ELISA for total glucagon (R&D, cat no. DY1249) and normalized by cell number. Peptidase inhibitors were not used in any of the studies.

### Statistics and data reproducibility

Data are presented as mean ± standard error of the mean (SEM), with each data point representing an independent biological replicate. All statistical analyses were performed using GraphPad Prism version 10.2.1. Where sample sizes permitted, normality was assessed using the Shapiro–Wilk test and visual inspection of Q-Q plots. For studies with small sample sizes, formal tests of normality were not performed because such tests have limited power to reliably characterize the underlying data distribution. Instead, data distributions and individual data points were visually examined before analysis. Statistical comparisons were performed using two-way analysis of variance (ANOVA), paired two-tailed t-tests, or unpaired two-tailed t-tests, as appropriate. Nonparametric tests were used when the assumptions required for parametric analysis could not be reasonably supported. Statistical significance was defined as *P* < 0.05 and is indicated as follows: ns, *P* ≥ 0.05; *, *P* < 0.05; and **, *P* < 0.01. Single-cell RNA-sequencing data were analyzed using the Wilcoxon rank-sum test, with the Holm–Bonferroni method used to adjust for multiple comparisons. The specific statistical tests and sample sizes used for each experiment are provided in the corresponding figure legends.

## Data Availability

The datasets presented in this study can be found in the GEO database at https://www.ncbi.nlm.nih.gov/geo/, under the accession code GSE138857.
