# Supplementary material for: CD98hc marks a precursor of human stem cell-derived alpha cells primed for functional maturation
Source: Front Endocrinol (Lausanne). 2026 Sep 28;17:1923474. doi: 10.3389/fendo.2026.1923474 (PMC13654763; doi:10.3389/fendo.2026.1923474)
Supplement: Supplementary file 1 [file DataSheet1.pdf]

Supplemental Figure 1:

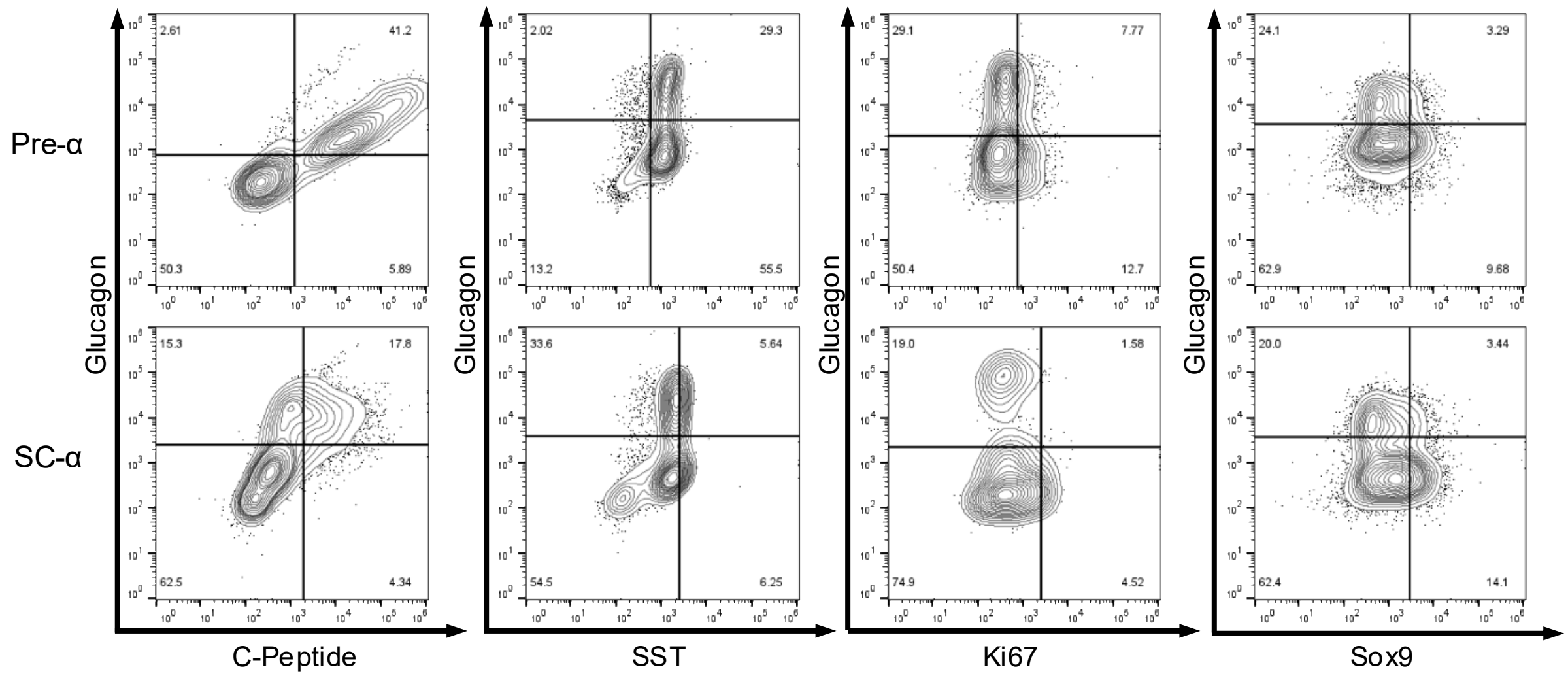

Supplemental Figure 1: Side Populations Present During Pre- and SC-α Stages.
Representative cell population flow cytometry present in the pre- and SC-α cell stages.

Supplemental Figure 2:

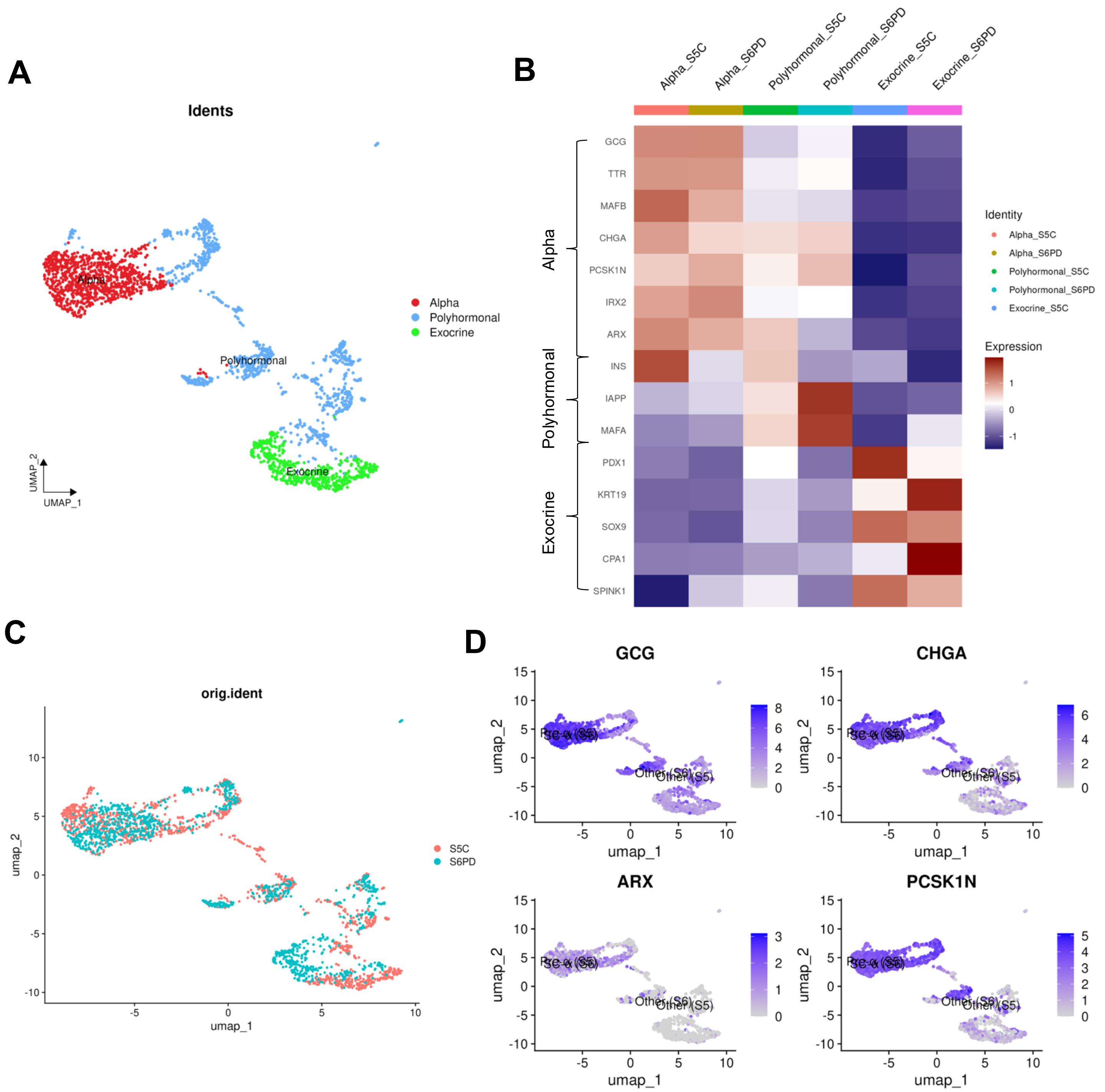

Supplemental Figure 2: Side Populations Present During Pre- and SC-α Stages.
A) Pre-α (S5C) and SC-α (S6PD) datasets clustered based on transcriptional identity.
B) Heatmap used to identify clusters from panel B.
C) Pre-α (S5C) and SC-α (S6PD) datasets shown separately from the pooled analysis in Fig 1C. Distribution of “Alpha” and “Other” populations are similar between the two datasets.
D) Feature plots of necessary α cell genes colocalizing with the alpha cluster in both pre- and SC-α stage data.

Supplemental Figure 3:

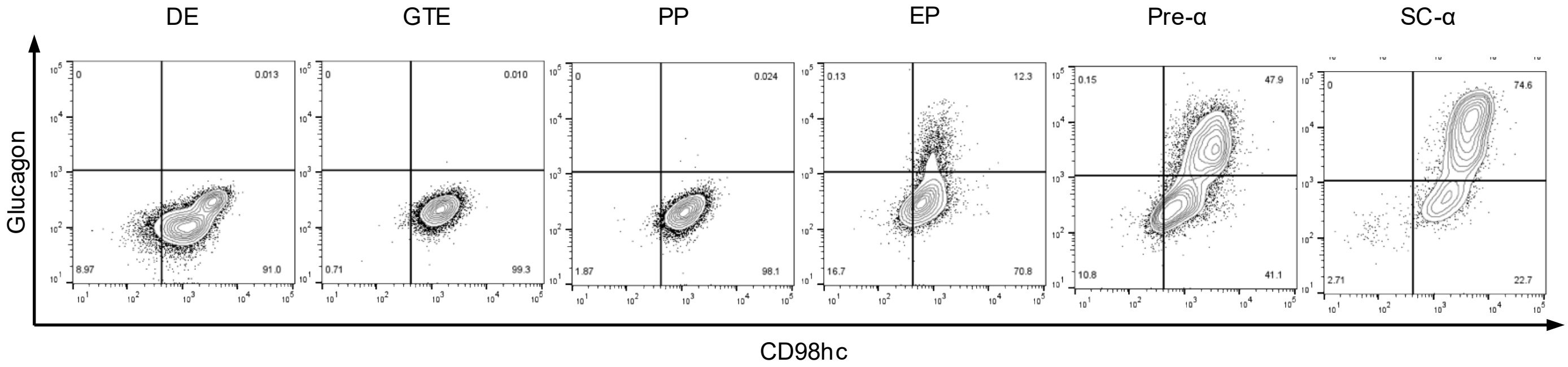

Supplemental Figure 3: Original Flow Cytometry Data From Main Figure 2
Flow cytometry plot with original axes present from main figure 2 (same experiment and FCS files). Stages are abbreviated as follows: definitive endoderm (DE), gut tube endoderm (GTE), pancreatic progenitor (PP), endocrine progenitor (EP).

Supplemental Figure 4:

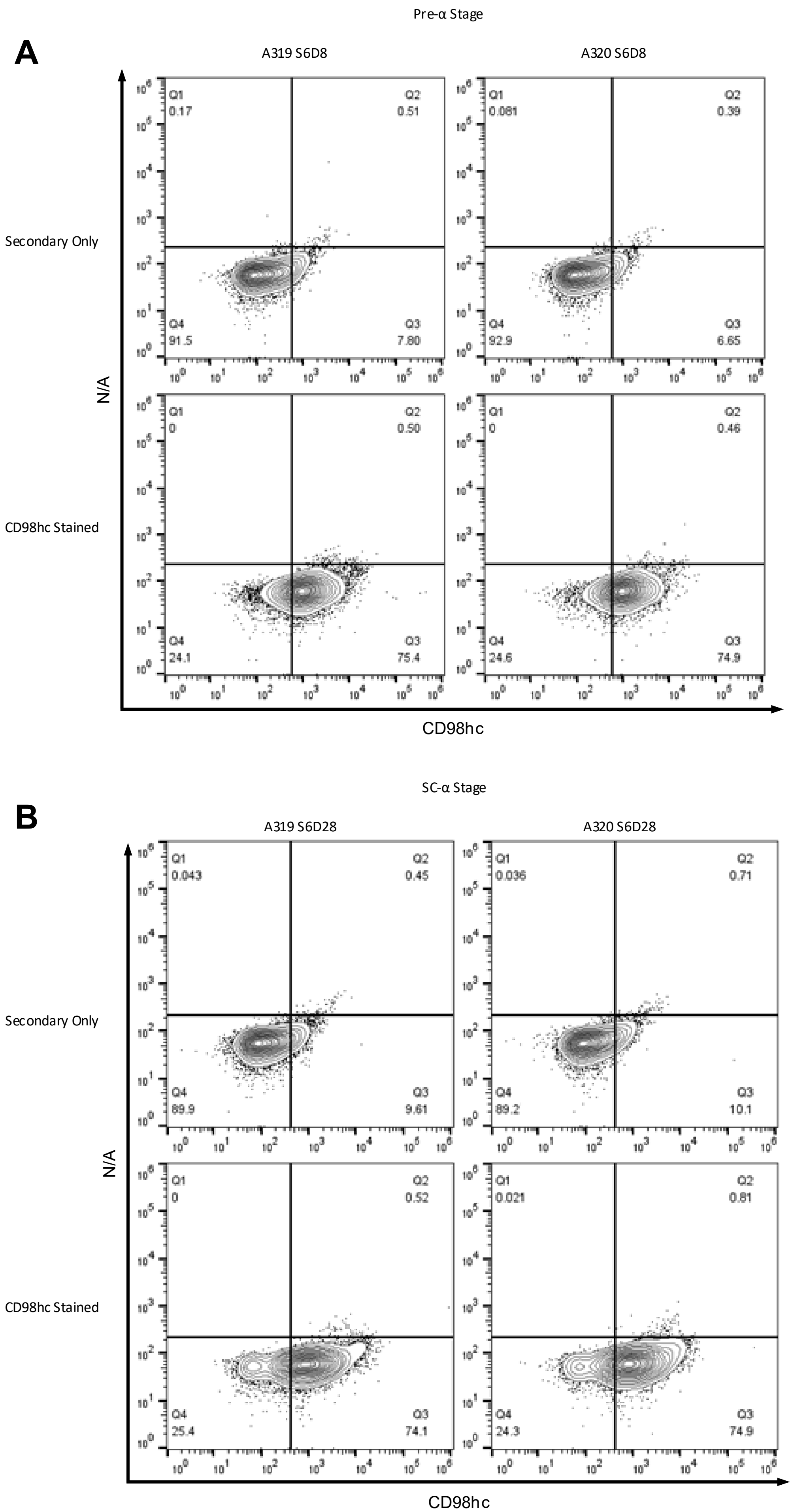

Supplemental Figure 4: Live cell staining of Pre- and SC-α stage cells for CD98hc.
Flow cytometry plots demonstrating that both live cell stained pre-α (A) and SC-α (B) cells express CD98hc protein on the cell surface.

Supplemental Figure 5:

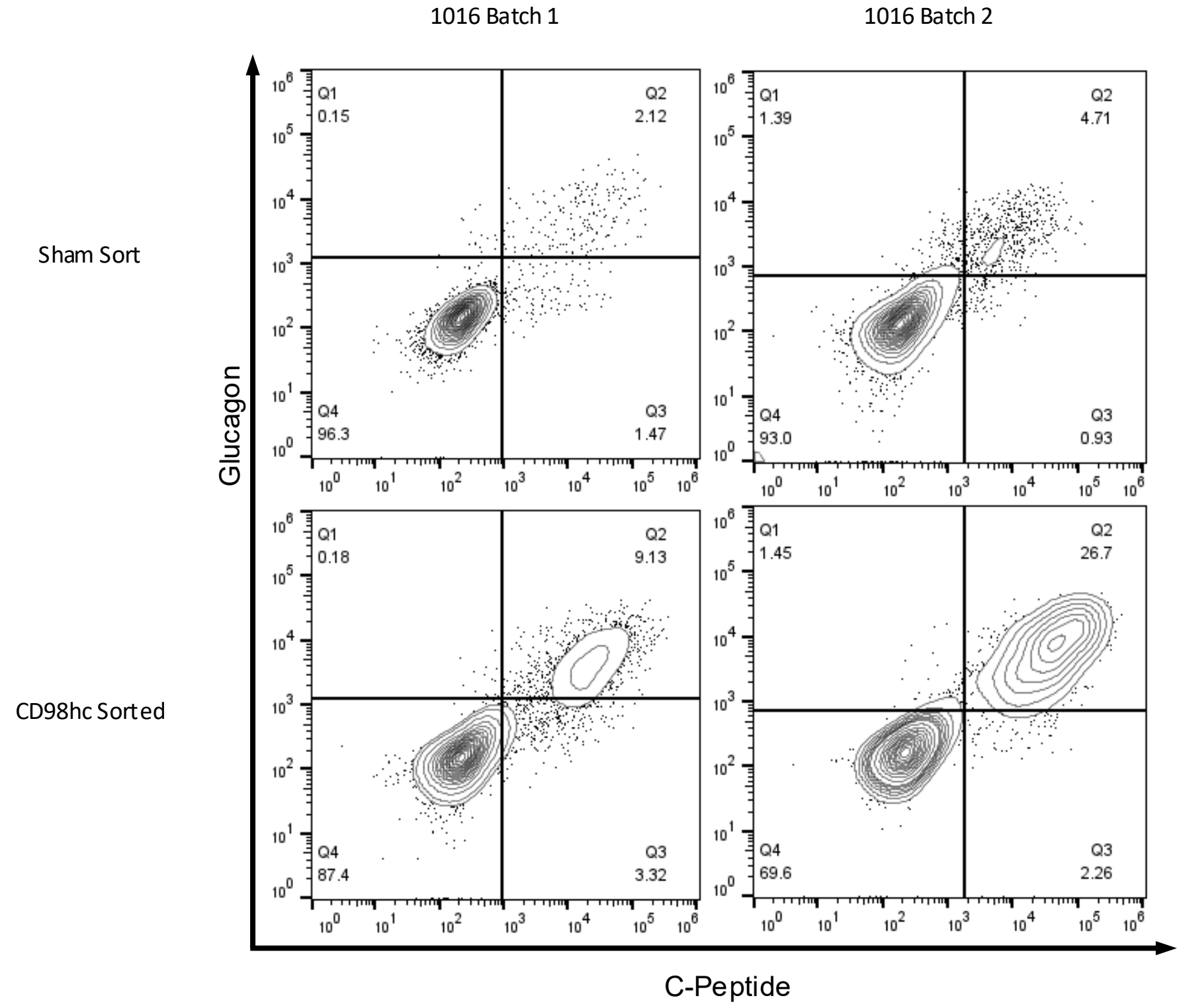

Supplemental Figure 5: Enrichment of pre-alpha cells derived from the 1016 iPS cell line. Flow cytometry plot demonstrating the unenriched and enriched populations in cells differentiated from the 1016 iPS cell line. CD98hc based enrichment of pre-α cells is effective in the 1016 iPS cell line demonstrating that CD98hc based enrichment is not cell line specific.

Supplemental Figure 6:

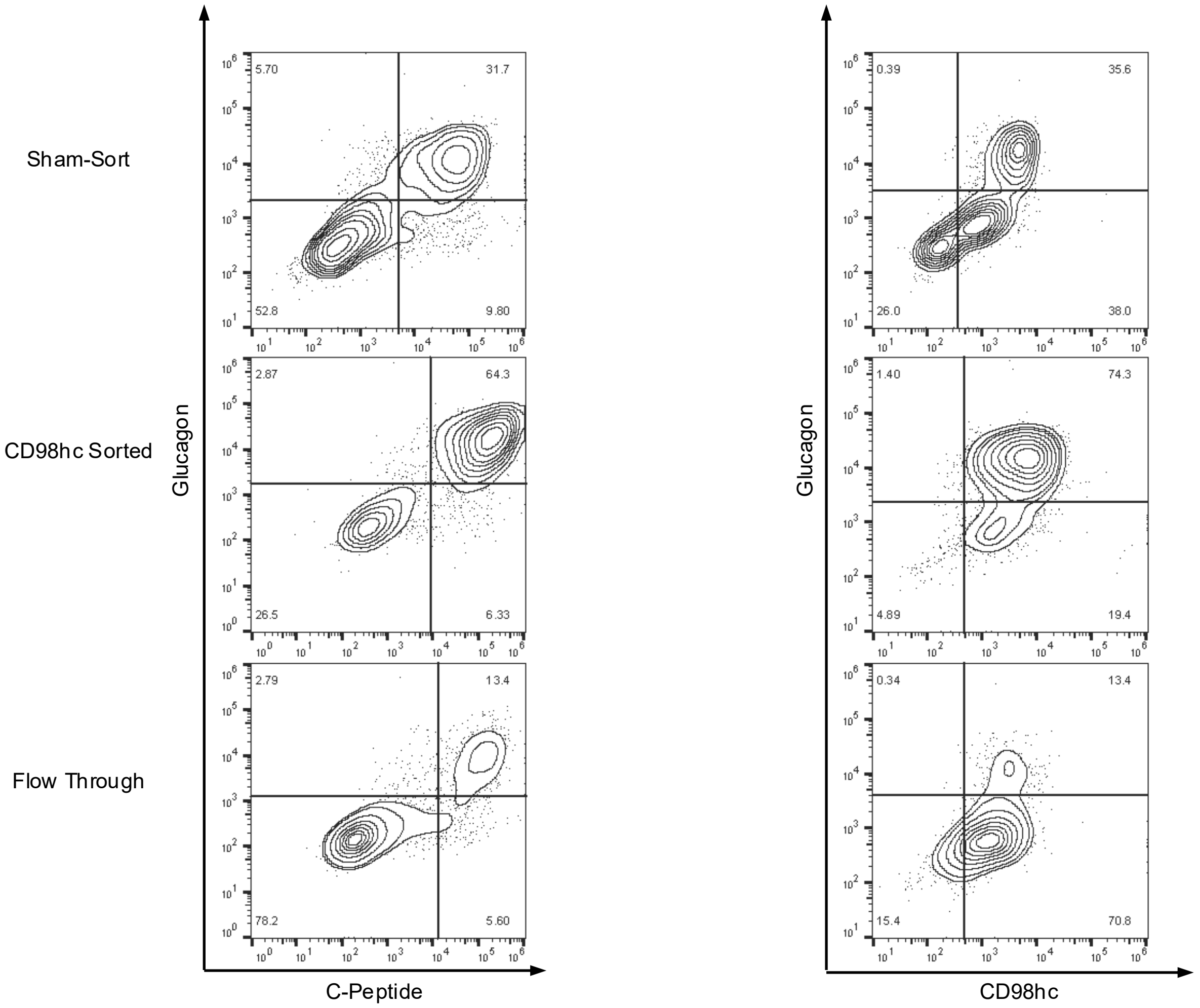

Supplemental Figure 6: Original Flow Cytometry Data From Main Figure 3
Flow cytometry plot with original axes present from main figure 3 (same experiment and FCS files).

Supplemental Figure 7:

A

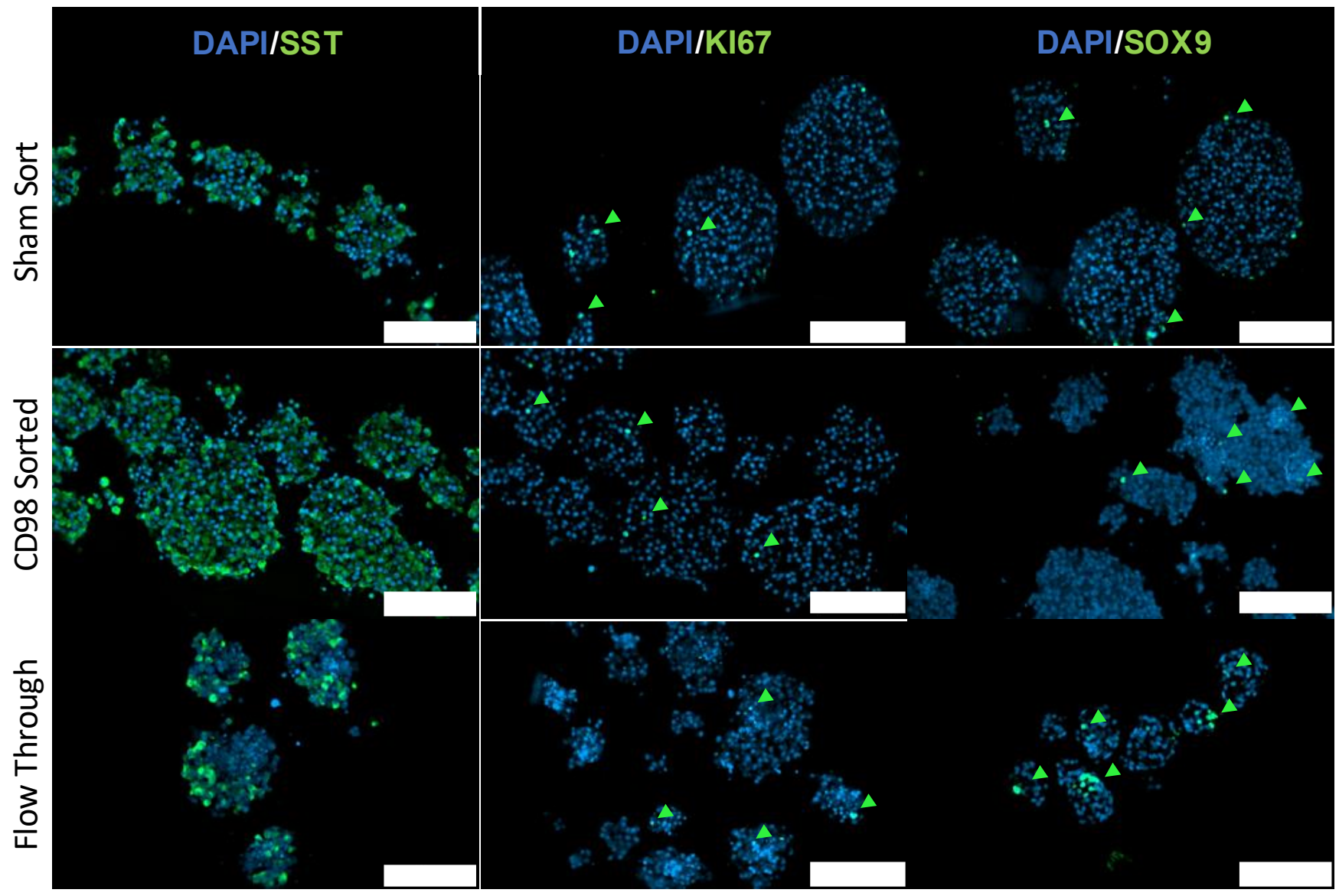

B

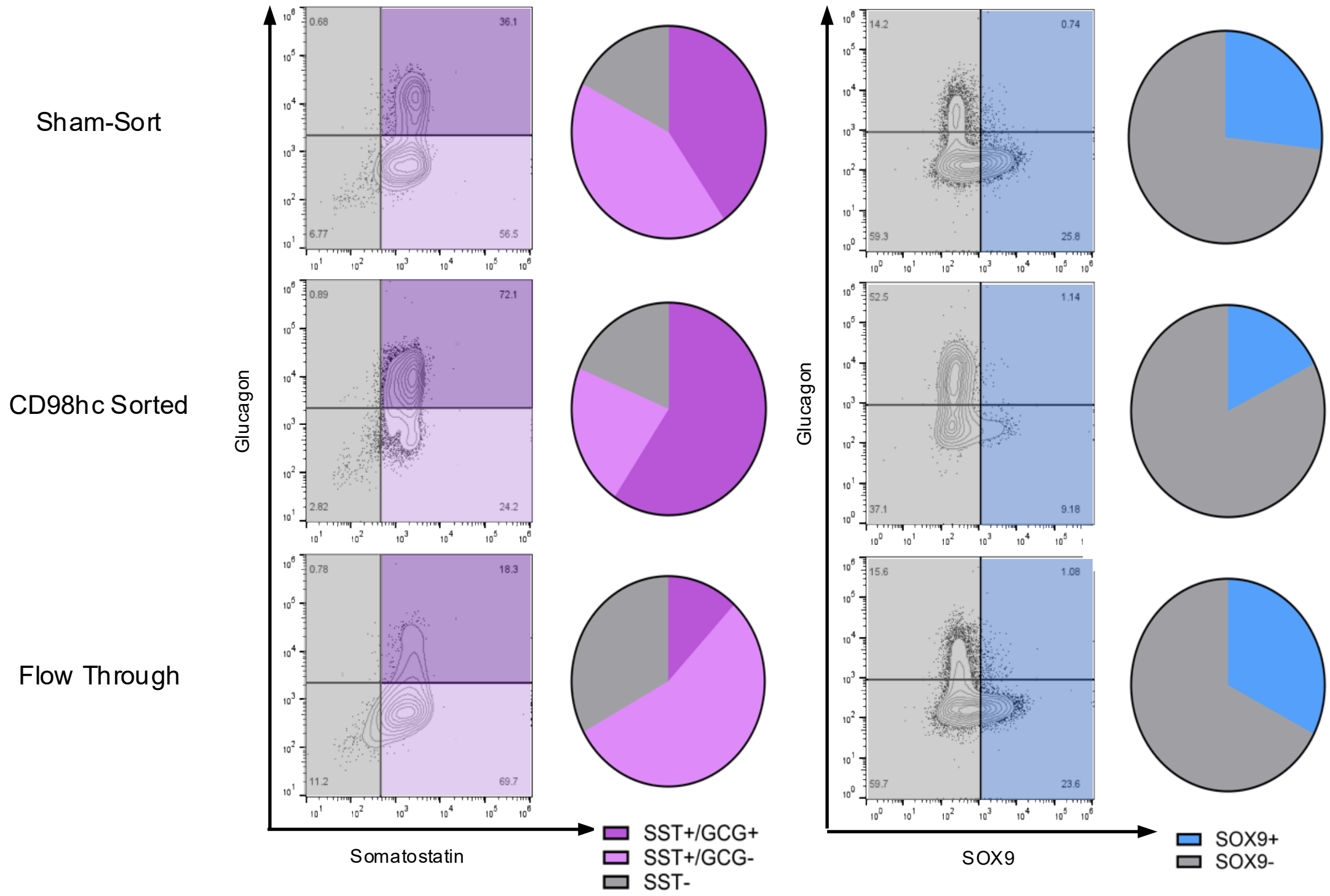

Supplemental Figure 7: Additional Cell Populations Assessed Post Enrichment.

A) Additional cell populations assessed post enrichment via immunofluorescence.

B) Somatostatin and Sox9 stained fractions following CD98 based enrichment representative (left) and multiple flow cytometry experiments averaged together (right) (n=7 independent experiments).

Supplemental Figure 8:

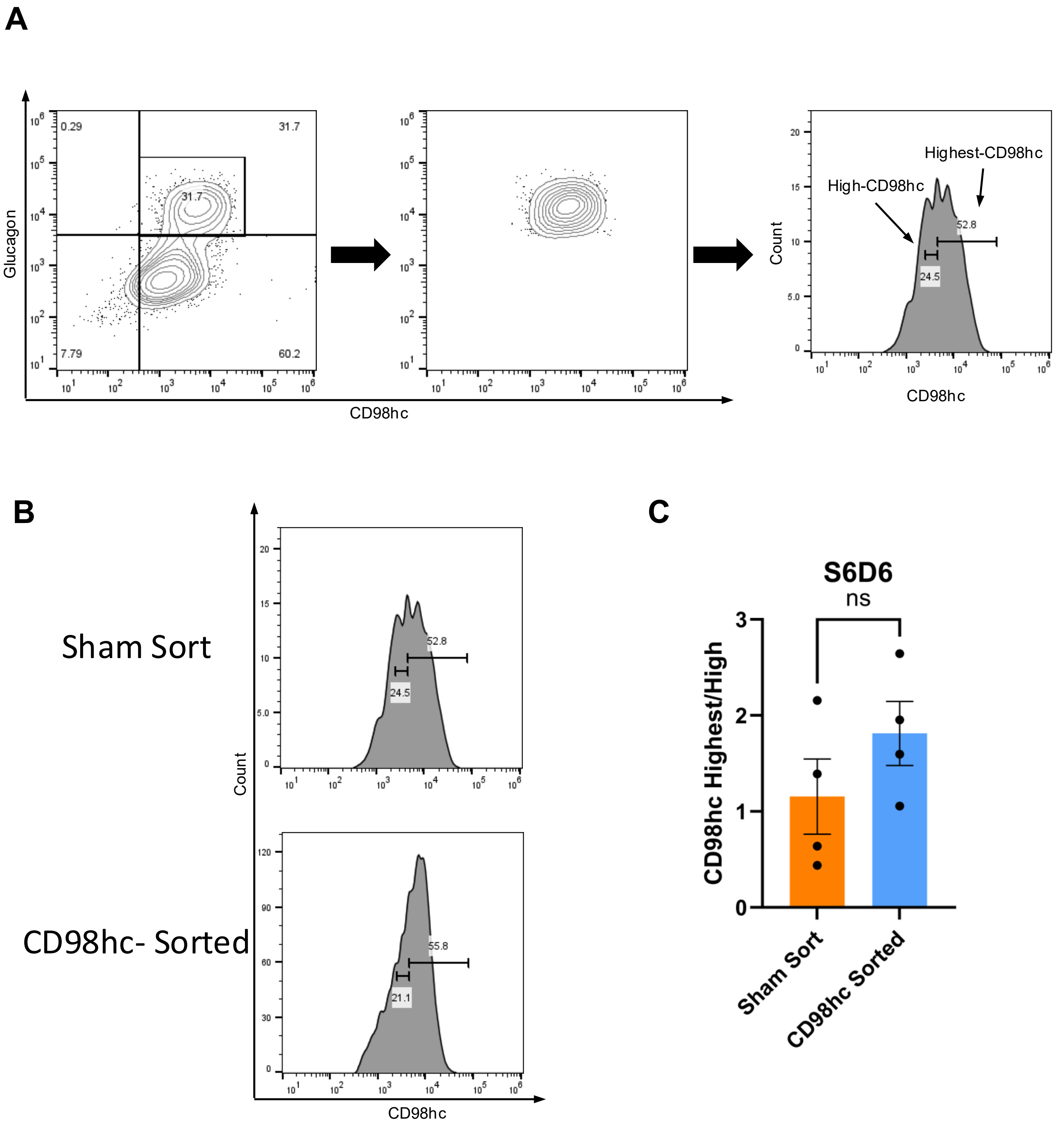

Supplemental Figure 8: Highest-CD98hc expressors relative to high-CD98hc expressors analysis

A) Example gating strategy to identify "highest-CD98hc" and "high-CD98hc" subpopulations within the CD98hc+/GCG+ population.
B) Representative histograms from sham sort and CD98hc-sorted fractions following enrichment with corresponding high- and highest-CD98hc subpopulations.
C) Bar graph depicting the ratios of highest-CD98hc relative to high-CD98hc subpopulation percentages across many enrichments (n=4 biological replicates) analyzed by paired t-test.

Supplemental Figure 9:

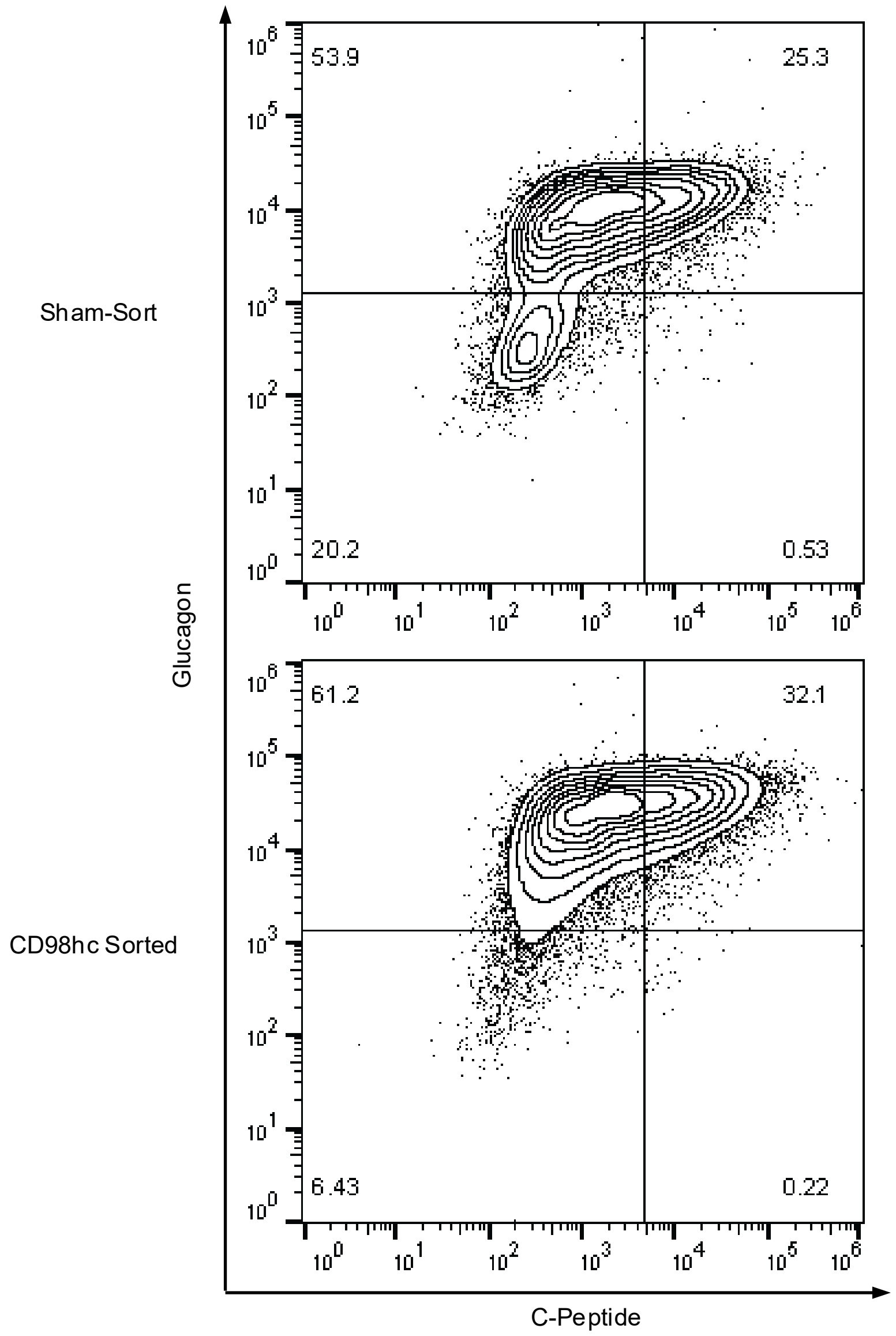

Supplemental Figure 9: Original Flow Cytometry Data From Main Figure 4
Flow cytometry plot with original axes present from main figure 4 (same experiment and FCS files).

Supplemental Figure 10:

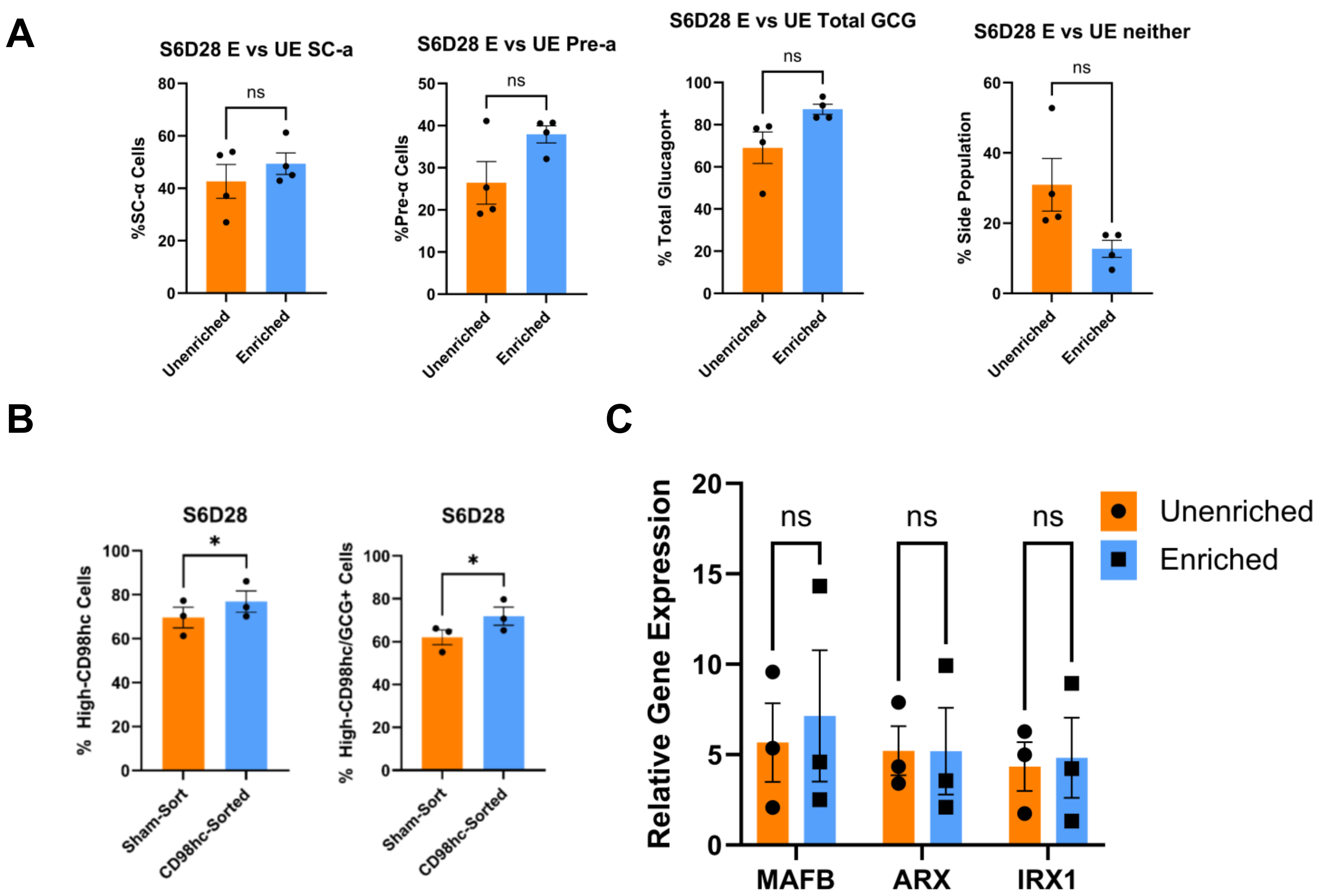

Supplemental Figure 10: Similarities Between SC-α Stage Sham- and CD98hc Sorted Populations.

A) Cell population percentages from flow cytometry data. No populations are significantly different between unenriched and enriched SC-α cells (n=4 independent experiments), analyzed by paired t-test.

B) High-CD98hc population percentages without and with glucagon co-positivity in SC-α cells derived either from sham-sorted controls or CD98hc sorted pre-α cells (n=3 independent experiments), analyzed by paired t-test *p<0.05.

C) Quantitative PCR data of α-cell identity genes performed on enriched and unenriched SC-α cells. There is no significant difference between the groups (n=3 biological replicates) analyzed by 2way ANOVA.

Supplemental Figure 11:

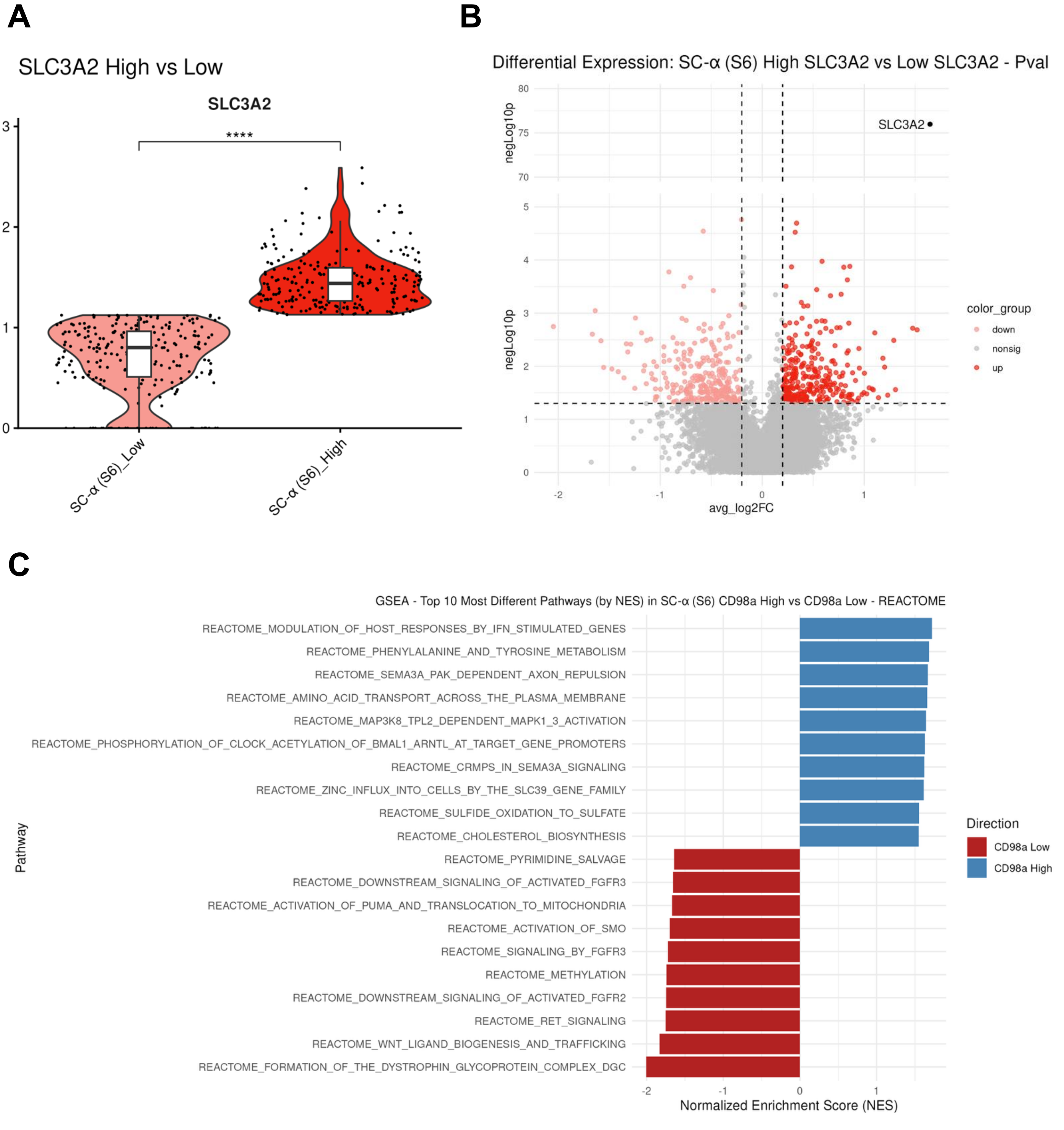

Supplemental Figure 11: Transcriptomic maturity of high-CD98hc expressing cells within the alpha cluster

A) Violin plot of “alpha” cluster cells with higher and lower than median levels of expression of SLC3A2 (CD98hc) Wilcox t-test, and Holm-Bonferroni multiple comparisons adjustment method.
B) Differential expression analysis performed on “high” expressors relative to “low” expressors of SLC3A2 (log2 fold change>0.2, p<0.05).
C) Pathway analysis of single cell RNA sequencing data of CD98hc high and CD98hc low populations demonstrate enrichment in mature alpha cell pathways including amino acid metabolism and transport, circadian regulation and zinc transport.

Supplemental Table 1. List of primers used for qPCR

Genes	Forward Primer	Reverse Primer
MAFB	AGA CGC CTA CAA GGT CAA GTG C	CGA CTC ACA GAA AGA ACT CGG G
IRX1	TCT CAG CCT CTT CTC GCA GAT G	GGT CCC CGT ATT GGA ACT GGC
ARX	CTG CTG AAA CGC AAA CAG AGG C	CTC GGT CAA GTC CAG CCT CAT G
GAPDH	ATC ACC ATC TTC CAG GAG CGA	TTC TCC ATG GTG GTG AAG ACG

Supplemental Table 2. List of Primary and Secondary Antibodies used for Flow cytometry and Immunofluorescence

Antibody	Species	Vendor	Catalog Number	Dilutions
Glucagon	Mouse	Millipore-Sigma	G2654	(1:1000 Flow/IF)
Glucagon	Rabbit	LS-Bio	LSB10219	(1:10,000 Flow/1:1000 IF)
C-Peptide	Rat	Developmental Sciences Hybridoma Bank	GNID4-S	(1:250 Flow/IF)
Somatostatin	Mouse	Santa Cruz	SC-25262	(1:250 Flow)
Somatostatin	Rabbit	Novus Biologicals	NBP1-87022	(1:100 IF)
Ki67	Mouse	Abcam	Ab238020	(1:500 Flow)
Ki67	Rabbit	R&D	MAB7617	(1:100 IF)
Oct4	Mouse	Santa Cruz	SC-5279	(1:250 Flow/1:2000 IF)
Sox9	Rabbit	Abcam	Ab185966	(1:250 Flow/IF)
Chromogranin A	Rabbit	Abcam	Ab283265	(1:2000 flow/1:2000 IF)
CD98	Mouse	Invitrogen	MA1-19195	(1:1000 Flow)
CD98	Rabbit	Proteintech	15193-1-AP	(1:5000 IF)
Sox17	Goat	R&D	AF1924	(1:250 Flow)
HNF1β	Mouse	Santa Cruz	SC-130407	(1:100 Flow)
PDX1	Goat	R&D	AF2419	(1:250 Flow)
NKX6.1	Mouse	Developmental Sciences Hybridoma Bank	F55A12	(1:100 Flow)
Alexa-488	Anti-Rabbit	Invitrogen	A21206	(1:1000 Flow/IF)
Alexa-488	Anti-Rat	Invitrogen	A21208	(1:1000 Flow/IF)
Alex-488	Anti-Mouse	Invitrogen	A21202	(1:1000 Flow/IF)
Alexa-594	Anti-Rabbit	Invitrogen	A21207	(1:1000 Flow/IF)
Alexa-594	Anti-Rat	Invitrogen	A21209	(1:1000 Flow/IF)
Alexa-594	Anti-Mouse	Invitrogen	A21203	(1:1000 Flow/IF)
Alexa-647	Anti-Rabbit	Invitrogen	A31573	(1:1000 Flow/IF)
Alexa-647	Anti-Rat	Invitrogen	A48272	(1:1000 Flow/IF)
Alexa-647	Anti-Mouse	Invitrogen	A31571	(1:1000 Flow/IF)
